# MYCN acts as a direct co-regulator of p53 in MYCN amplified neuroblastoma

**DOI:** 10.18632/oncotarget.24859

**Published:** 2018-04-17

**Authors:** Saurabh Agarwal, Giorgio Milazzo, Kimal Rajapakshe, Ronald Bernardi, Zaowen Chen, Eveline Barberi, Jan Koster, Giovanni Perini, Cristian Coarfa, Jason M. Shohet

**Affiliations:** ^1^ Division of Hematology-Oncology, Department of Pediatrics, Baylor College of Medicine, Houston, Texas, USA; ^2^ Department of Pharmacy and Biotechnology, University of Bologna, Bologna, Italy; ^3^ Dan L Duncan Cancer Center, Department of Molecular and Cellular Biology, Baylor College of Medicine, Houston, Texas, USA; ^4^ Department of Oncogenomics, Academic Medical Center, University of Amsterdam, Amsterdam, The Netherlands

**Keywords:** neuroblastoma, MYCN, p53, p53 C-terminal domain, DNA damage resposone

## Abstract

The MYC oncogenes and p53 have opposing yet interrelated roles in normal development and tumorigenesis. How MYCN expression alters the biology and clinical responsiveness of pediatric neuroblastoma remains poorly defined. Neuroblastoma is p53 wild type at diagnosis and repression of p53 signaling is required for tumorigenesis. Here, we tested the hypothesis that MYCN amplification alters p53 transcriptional activity in neuroblastoma. Interestingly, we found that MYCN directly binds to the tetrameric form of p53 at its C-terminal domain, and this interaction is independent of MYCN/MAX heterodimer formation. Chromatin analysis of MYCN and p53 targets reveals dramatic changes in binding, as well as co-localization of the MYCN-p53 complex at p53-REs and E-boxes of genes critical to DNA damage responses and cell cycle progression. RNA sequencing studies show that MYCN-p53 co-localization significantly modulated the expression of p53 target genes. Furthermore, MYCN-p53 interaction leads to regulation of alternative p53 targets not regulated in the presence of low MYCN levels. These novel targets include a number of genes involved in lipid metabolism, DNA repair, and apoptosis. Taken together, our findings demonstrate a novel oncogenic role of MYCN as a transcriptional co-regulator of p53 in high-risk MYCN amplified neuroblastoma. Targeting this novel oncogenic function of MYCN may enhance p53-mediated responses and sensitize MYCN amplified tumors to chemotherapy.

## INTRODUCTION

Neuroblastoma accounts for almost 15% of all pediatric cancer mortality. In approximately 50% of high-risk cases (25% overall), MYCN is amplified with between 10 to 500 additional copies of this oncogene (as double minutes or HSRs) and up to 10,000 copies of MYCN mRNA leading to very high levels of the MYCN protein. Neuroblastoma tumors with low levels of MYCN have better overall responses to chemotherapy, significantly better overall survival, and higher response rates to second line chemotherapy regimens [1, 2]. MYCN amplified tumors also tend to relapse earlier and rapidly develop chemotherapy resistance [3]. Patients with relapsed MYCN amplified tumors have less than 5% overall survival.

Despite its well-defined negative impact on clinical outcome, the mechanisms distinguishing the clinical behavior of MYCN amplified and non-amplified neuroblastoma remain poorly defined. Indeed, multiple studies have sought to define predictive prognostic signatures to identify patients for more intensive or alternative treatment [4, 5]. Intriguingly, most published prognostic gene signatures have little overlap and the primary risk factor for poor outcome remains MYCN expression levels. Recently, a combinatorial analysis of these signatures highlighted a common ‘MYCN’ module enriched for deregulation of DNA damage repair genes [6]. Overall, the primary factor distinguishing high-risk tumors and predicting overall survival remains MYCN amplification itself.

MYCN is a bHLH transcription factor closely related to C-Myc. This oncogene binds to E-box sequences as a heterodimer with its cognate binding partner MAX. Deregulated persistent expression of MYCN plays critical role in neuroblastoma pathogenesis and is a well-defined driver of tumor initiation from early neural crest precursors [7, 8]. Here specific transcriptional targets such as MDM2, ODC1, phox2B, and others contribute to early developmental arrest and tumorigenesis. In addition, the function of MYCN/MAX and C-Myc/MAX heterodimers as global transcriptional amplifiers regulated by super-enhancer elements likely also plays a major role in neuroblastoma development. However, these studies do not explain the clinical observation of markedly higher early treatment failures for MYCN-amplified compared to non-amplified tumors (20% compared 0% in one study) [3]. Nor that MYCN amplified tumors are more likely to develop drug resistant disease and relapse soon after stopping therapy [9]. Indeed, MAX is not co-amplified with MYCN, suggesting the novel hypothesis that alternative MYCN functions may account for the aggressive biology of MYCN-amplified disease.

Another important observation is that *de novo* neuroblastoma is uniformly p53 wild-type at diagnosis (>98% by DNA sequencing) [10]. This is of particular interest for a Myc driven tumor as both MYCN and C-Myc alter DNA damage responses, override cell cycle checkpoints, drive proliferation, and alter metabolic pathways. Together these can induce cellular stress responses that provoke p53 dependent apoptosis. Upon activation, p53 forms dimers and which associate to form tetramers that bind to promoter DNA at p53 response elements (p53-REs). Multiple studies demonstrate that the specificity and affinity of this activity is modulated by damage induced acetylation and other post-translational modifications of the C-terminal regulatory domain (CTD) [11–13]. Mutations and deletions of the p53 CTD are linked to several cancers and lead to p53 binding and activation of divergent target genes [12]. A recent study demonstrated that binding of the oncoprotein SET to the non-acetylated CTD strongly suppresses p53 signaling [14]. In addition, the histone demethylase LSD1 specifically removes mono- or di-methylation at K370 of p53 to repress p53-mediated transcriptional activation [15]. Several studies also demonstrate protein/protein interactions of C-Myc or MYCN (together referred to as Myc(N)) with LSD1, that alter expression of critical cancer related genes [16, 17].

Based on the observed extreme stoichiometry of MYCN in MYC-amplified tumors, and their distinct response to chemotherapy, we hypothesized that non-canonical MYCN activities contribute to their aggressive phenotype and poor clinical outcomes. Here we demonstrate a direct protein/protein interaction for Myc(N) and the p53 CTD. This interaction alters binding of both transcription factors at promoter binding sites and deregulates the transcriptional response to genotoxic stress. We demonstrate nuclear co-localization and binding of MYCN with p53 in response to MDM2 inhibition exclusively in cells with very high MYCN expression. Gene expression studies further demonstrate altered expression of a number of novel stress response genes exclusively modulated under MYCN amplified conditions. These include genes controlling lipid metabolism, DNA damage, and oncogenic signaling. Most of these genes correlate with significantly worse overall survival in neuroblastoma patient cohorts. Together these data are consistent with a model where MYCN acts as a co-factor with p53, altering both MYCN and p53 transcriptional responses independent of MYCN/MAX mediated DNA binding. We further discuss the biological and clinical implications of these findings.

## RESULTS

### MYCN and p53 co-localize under amplified conditions

**Figure 1:**
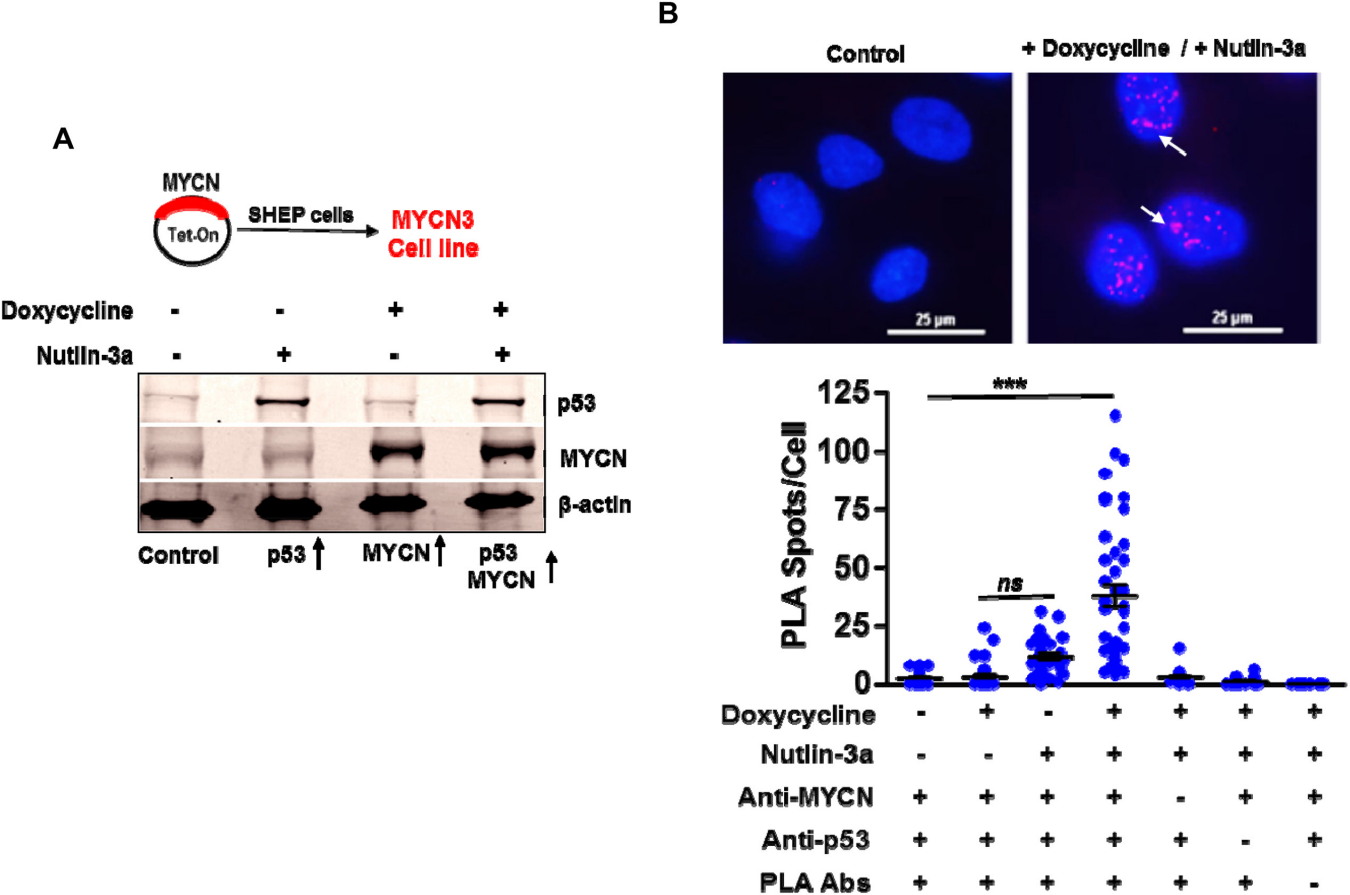
MYCN and p53 co-localize and bind to each other **(A)** The MYCN3 cell line was generated by transfecting a Tet-On plasmid containing full-length MYCN cDNA. MYCN3 cells were treated with Doxycycline to induce MYCN levels and with Nutlin-3a to induce p53 levels. Western blot showing MYCN and p53 protein levels under different treatment conditions. **(B)** Proximity ligation assays (PLA) for MYCN and p53 binding were performed using MYCN3 cells in the presence or absence of doxycycline and Nutlin-3a treatments. Additional controls were performed to determine antibody specificity. Representative images of control and combined doxycycline and Nutlin-3a treated cells are shown. A Scatter-plot and the mean ± SEM of the number of PLA spots per cell are shown in the bottom panel. The *p*-values of the difference between combination treatments and all other groups was <0.05 (Mann-Whitney test). Arrows indicating prominent PLA spots. ^***^ p<0.001, ns= non significant.

To evaluate non-canonical MYCN functions we first tested the hypothesis that MYCN and p53 closely associate when p53 is activated in the presence of high levels of MYCN. We used a MYCN inducible system (Tet-ON) in p53 wild-type SHEP neuroblastoma cells to model these conditions. To avoid interference from fusion tags, we used a conditional construct expressing wild type MYCN with no 5’ or 3’ tags [18]. These cells stabilize and activate p53-mediated responses in response to Nutlin-3a (an MDM2 inhibitor) as shown in Figure 1A. Next, we used proximity ligation assays (PLA) to evaluate interactions of endogenous MYCN with p53. As shown, focal nuclear fluorescence signals of MYCN and p53 proximity interactions significantly increased only when MYCN and p53 were both present at high levels (Figure 1B). Of note, little to no PLA signals were detected in controls or when p53 is activated under non-MYCN conditions (Figure 1B).

### MYCN and p53 are direct binding partners

**Figure 2:**
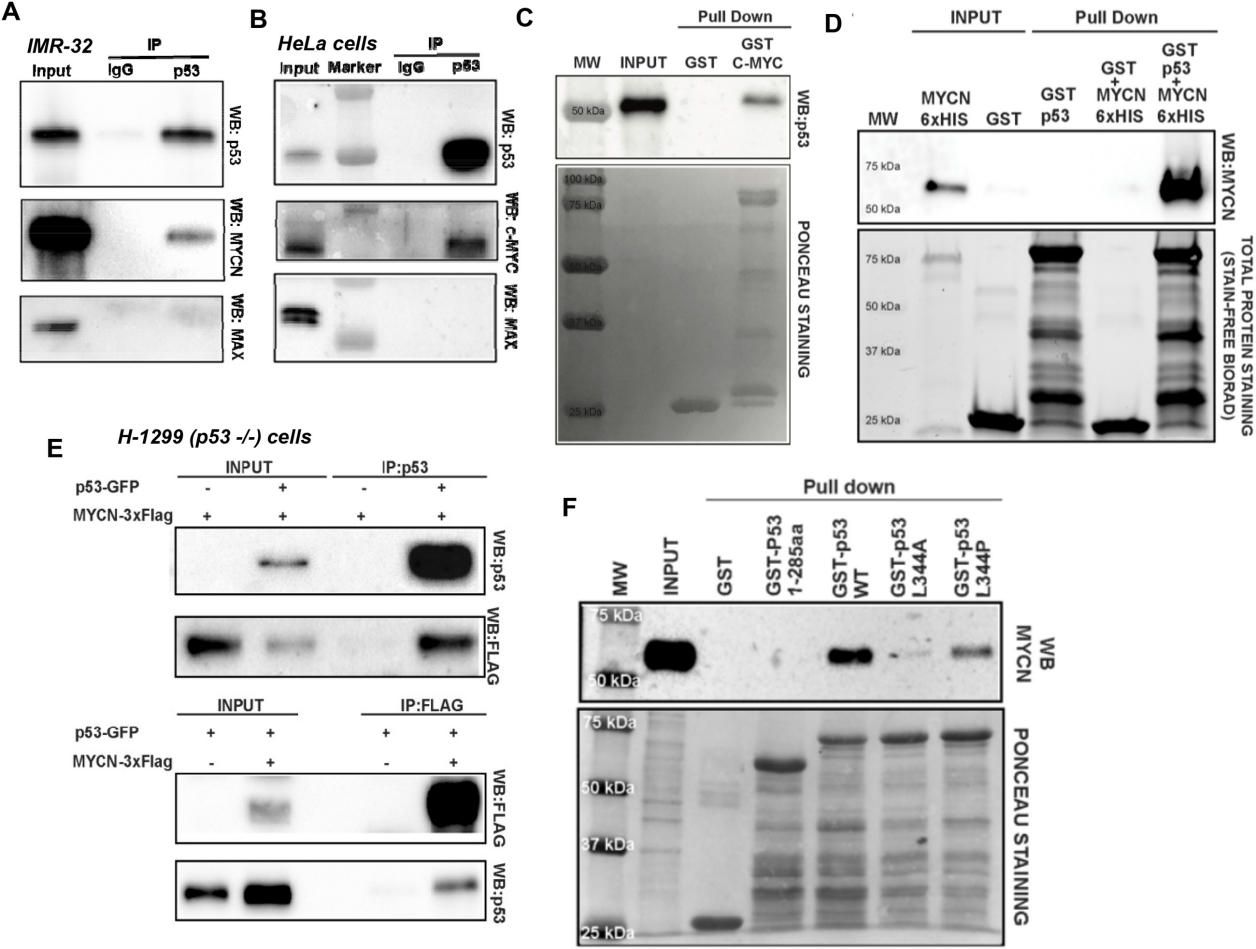
**(A)** Endogenous MYCN and p53 co-IP. Nuclear extracts from the neuroblastoma cell line IMR-32 treated with Nutlin-3a were co-immunoprecipitated using anti-p53 (Ab-7) antibody or IgG (negative control). Western blots of immunoprecipitated proteins were performed using anti-p53 (DO-1), anti- MYCN (B8.4.B), or anti-Max (C-17) antibodies. **(B)** Endogenous MYC and p53 co-IP. HeLa cells treated with Nutlin-3a were co-immunoprecipitated using anti-p53 antibody or negative control IgG. Immunoprecipitated proteins were analyzed by Western blotting, using with anti-p53 (DO-1), anti- MYC (N262), and anti-MAX (C-17) antibodies. **(C)** *in vitro* GST-C-MYC pull-down. Crude nuclear protein extract from transient p53 over-expressing HEK-293T cells was incubated overnight with full-length GST-MYC or GST control proteins immobilized on glutathione-agarose beads. Pull-down samples were immunoblotted with the anti-p53 antibody. Membrane Ponceau S staining is shown as a loading control. **(D)** MYCN and p53 *in vitro* pull-down. Purified recombinant MYCN-6×His, GST-p53 (full length), and GST-control proteins were loaded as input samples. Recombinant MYCN- 6×His protein was incubated with GST-p53 or GST-control proteins immobilized on glutathione-agarose beads. GST proteins were pulled down and associated MYCN was detected by Western Blotting. Stain-Free total protein staining was used as a loading control. **(E)** Recombinant p53 and MYCN co-immunoprecipitation. The p53-null, non-small cell lung carcinoma cell line H-1299 was transiently transfected with plasmids overexpressing p53-GFP and MYCN-3×Flag. Crude nuclear protein extract collected from cells cultured under different transfection conditions were immunoprecipitated (IP) with either anti-p53 (Ab-7) or anti-FLAG (M2) antibody, and Western blots were performed using either anti-FLAG (M2) or anti-p53 (DO-1) antibody. **(F)** MYCN interacts with tetrameric form of p53. Crude nuclear protein extracts from MYCN-amplified SK-N-BE2-(C) cells were incubated with GST alone and a series GST-p53 purified proteins: p53-WT (dimeric-tetrameric), p53-L344A (dimeric only) and p53-L344P (monomeric only). Input and pull-down samples were immunoblotted using anti-MYCN antibody and Ponceau staining was used as loading control.

As the PLA data suggested a close interaction of MYCN and p53 in our conditional system, we sought to validate these results by co-immunoprecipitation assays using both endogenous and recombinant GST and FLAG tagged proteins (Figure 2). We demonstrate robust association of p53 with both MYCN (Figure 2A) and C-Myc (Figure 2B). Importantly, we found that MAX, the primary transcriptional cofactor for MYC(N), was not associated with either MYCN or C-Myc protein pulled down with p53 (Figure 2A, 2B). Additionally, GST-C-Myc is also co-immunoprecipitated with p53, when incubated with cell free extract of HEK-293 cells overexpressing p53 (Figure 2C). We further verified direct protein/protein interaction using an *in vitro* cell free system using purified recombinant MYCN-6×HIS and GST-p53 proteins. (Figure 2D). To evaluate recombinant protein interactions in a distinct and p53 null background (p53^-/-^), we used the non-small cell lung cancer cell line H1299 with transient transfection of p53-GFP and MYCN-3xFlag recombinant protein vectors. We again demonstrated robust interactions of MYCN with p53 by pulldown and Western blotting (Figure 2E). These data strongly suggest that both MYCN and C-Myc can directly bind to p53 when present at high levels and that MAX is not a component of this complex.

As p53 can be present in the cytoplasm and nucleus as monomers, dimers and tetramers, but only the tetrameric form binds to chromatin [19]. To determine which p53 state most actively bound to MYCN, we used two well-characterized mutants of p53: p53 L344A that forms dimer, but not tetramers, and p53 L344P, which is exclusively monomeric [20, 21]. The GST pulldown and MYCN Western blotting showed that MYCN bound to wild type tetrameric p53 with high affinity, to monomeric p53 with low affinity, and did not bind to dimeric p53 (Figure 2F).

### MYCN binds to the c-terminal of p53

**Figure 3:**
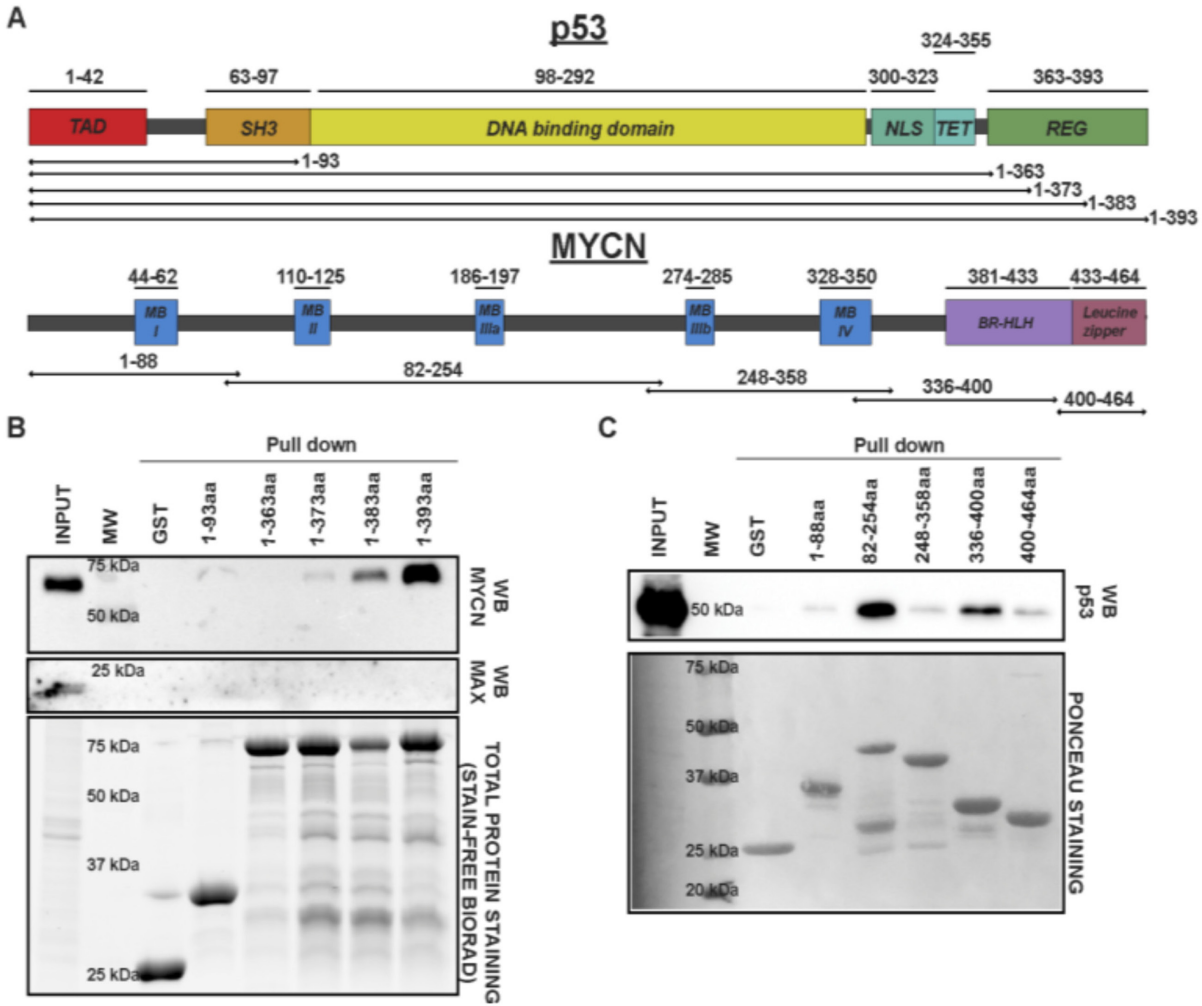
**(A)** Graphical representations of p53 and MYCN proteins. p53 (upper panel) and MYCN (lower panel) protein domains and truncation constructs. p53 protein domains: Trans Activation Domain (TAD), SRC Homology 3 domain (SH3), DNA binding domain, Nuclear Localization Signal (NLS), Tetramerization domain (TET), Regulatory domain (REG). MYCN protein domains: MYC boxes (MB), the basic region helix loop helix (BR-HLH), and the leucine zipper. The GST protein fragments are indicated with bars, and numbers refer to amino-acid positions. p53 and MYCN protein fragments were cloned in frame with the N-terminal GST in a pGEX-2T vector. GST-p53 and GST-MYCN fragments were cloned, expressed in BL-21 *E.Coli* strain and purified using gluthatione-agarose beads. **(B)** MYCN interacts with the C-terminus of p53. Crude nuclear protein extracts from MYCN-amplified SK-N-BE-(2)-c cells were incubated with the different p53 truncations or GST alone (negative control) immobilized onto glutathione-agarose beads. Input and pull-down samples were immunoblotted using anti-MYCN and anti-MAX antibodies. Stain-Free total protein staining was used as the loading control. **(C)** GST pull-down assay of MYCN truncations. Crude nuclear protein extract from transiently transfected p53-overexpressing HEK-293T cells was incubated with different MYCN-GST fragments immobilized on glutathione-agarose beads. GST alone was used as a negative control. Input and pull-down samples were immunoblotted using anti-p53 (DO-1) antibody. Ponceau staining was used as a loading control.

We next generated a series of p53- and MYCN- GST truncation mutants to map the respective interacting protein domains (Figure 3A). Nuclear extracts from MYCN-amplified SK-N-BE2-(C) neuroblastoma cells were incubated with different p53 truncations or GST alone. Interestingly, we find that the C-terminal 20 amino acids of p53 (aa 373-393) is required for MYCN interaction and pull down (Figure 3B). Next, crude extracts of HEK-293T transiently expressing full length p53 were incubated with different GST-MYCN truncation proteins. The results here were less specific likely due to the inherent disordered nature of MCYN protein, however, they suggest an interaction of p53 with the MycBOX II / IIIa domains, which are conserved between MYCN and C-Myc (Figure 3C). Taken together, these data clearly demonstrate a strong, context specific protein-protein interaction of MYCN or C-Myc with tetrameric form of p53 at c-terminal domain.

### MYCN binding significantly impacts p53 transcriptional responses

**Figure 4:**
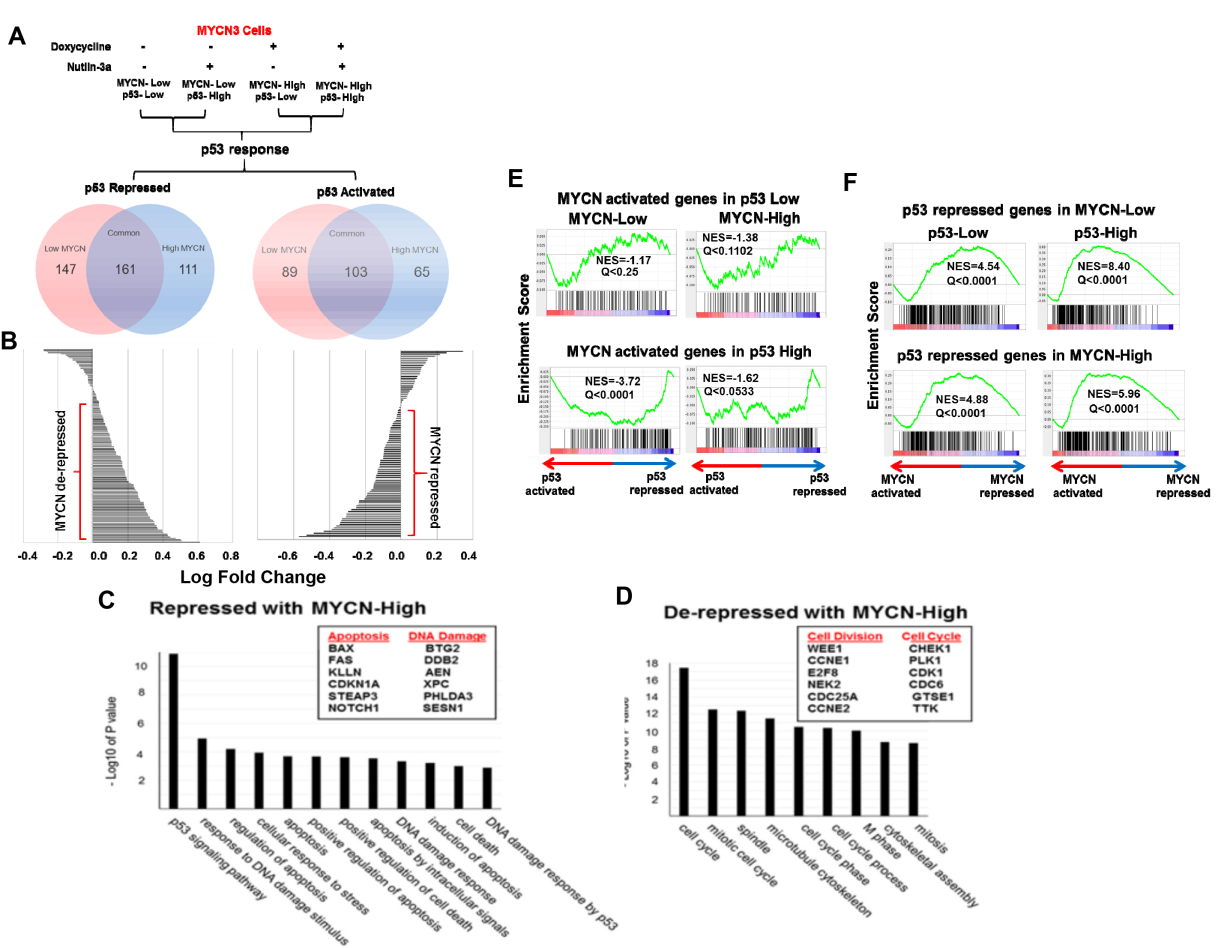
**(A)** RNA-Seq was performed on MYCN3 cells under different p53 and MYCN high conditions. Treatment conditions were compared to determine the effect of low and high MYCN levels on p53 response. p53 activated and repressed genes under high and low MYCN conditions were determined and shown with Venn diagrams. **(B)** Waterfall plots of the relative changes in gene expression of common genes shows the p53-regulated genes under low MYCN vs. high MYCN conditions. High MYCN levels relatively repressed the p53-up-regulated genes and relatively de-repressed the p53-down-regulated genes. **(C, D)** Functional annotation analysis of differentially expressed genes using the DAVID Bioinformatics platform. Representative genes analyzed are shown in boxes. **(E)** Gene Set Enrichment Analysis (GSEA) shows a statistically significant and robust interaction between the MYCN and p53 transcriptional programs. Genes induced by MYCN in either low or high p53 conditions are suppressed in the p53 transcriptional response regardless of MYCN levels (Normalized Enrichment Score [NES]<0, Q<0.25). **(F)** Genes repressed by p53 in either low or high MYCN conditions are induced in the MYCN transcriptional response regardless of p53 levels (NES>0, Q<0.25).

To understand the biological consequences of MYCN-p53 interactions in neuroblastoma, we performed unbiased genome-wide RNA-sequencing in the MYCN3 cell line, under all four different conditions of MYCN and p53 levels induced by doxycycline and Nutlin-3a treatments (Figure 4A) (GSE83328). To determine the p53 responses under MYCN-low and high conditions, we compared the two low MYCN conditions and two high MYCN conditions, as depicted in Figure 4A. The treatment conditions of control (no treatment, Doxycycline –, Nutlin3a -) was compared with MYCN low p53 high (Doxycycline -, Nutlin3a +) to determine the p53 response under MYCN low condition. Similarly, MYCN high p53 low (Doxycycline +, Nutlin3a -) was compared with MYCN high p53 high (Doxycycline +, Nutlin3a +) to determine p53 response under MYCN high condition. These p53 responses were analyzed to determine p53 repressed and p53 activated genes under MYCN low and high, as shown by van diagrams (Figure 4A). Under stringent conditions (fold change>1.3, FKPM>1), we found 161 p53 repressed and 103 activated genes that were common to both high and low MYCN conditions (Figure 4A). Interestingly, waterfall plot analysis of these 264 differentially regulated p53 response genes showed that high levels of MYCN opposed the p53 transcriptional activity. Genes typically up-regulated by p53 were repressed and those down-regulated by p53 were relatively de-repressed in the presence of high MYCN levels (Figure 4B). Functional annotation analysis of p53 responsive genes validated previous demonstrations that MYCN represses p53 apoptotic genes and increases proliferation and mitosis related genes (Figure 4C, 4D), Interestingly, we found a group of genes only modulated by p53 when MYCN levels were high. The top functional clusters of these genes included DNA metabolism, cell division, and chromosome segregation (Supplementary Figure 1-2) [22, 23]. These genes were not modulated by p53 in the absence of MYCN. Gene Set Enrichment Analysis (GSEA) of the genes revealed that activation of p53 was significantly correlated with lower MYCN transcriptional activity at a subset of MYCN target genes (Figure 4E; Q<0.25, normalized enrichment score [NES]<0). Conversely, activation of MYCN was significantly associated with increased transcriptional activity at genes repressed by p53 (Figure 4F, Q<0.25, NES>0). These data support a model in which MYCN generally opposes p53 functions.

### RNA-sequencing identifies non-canonical p53 targets

**Figure 5:**
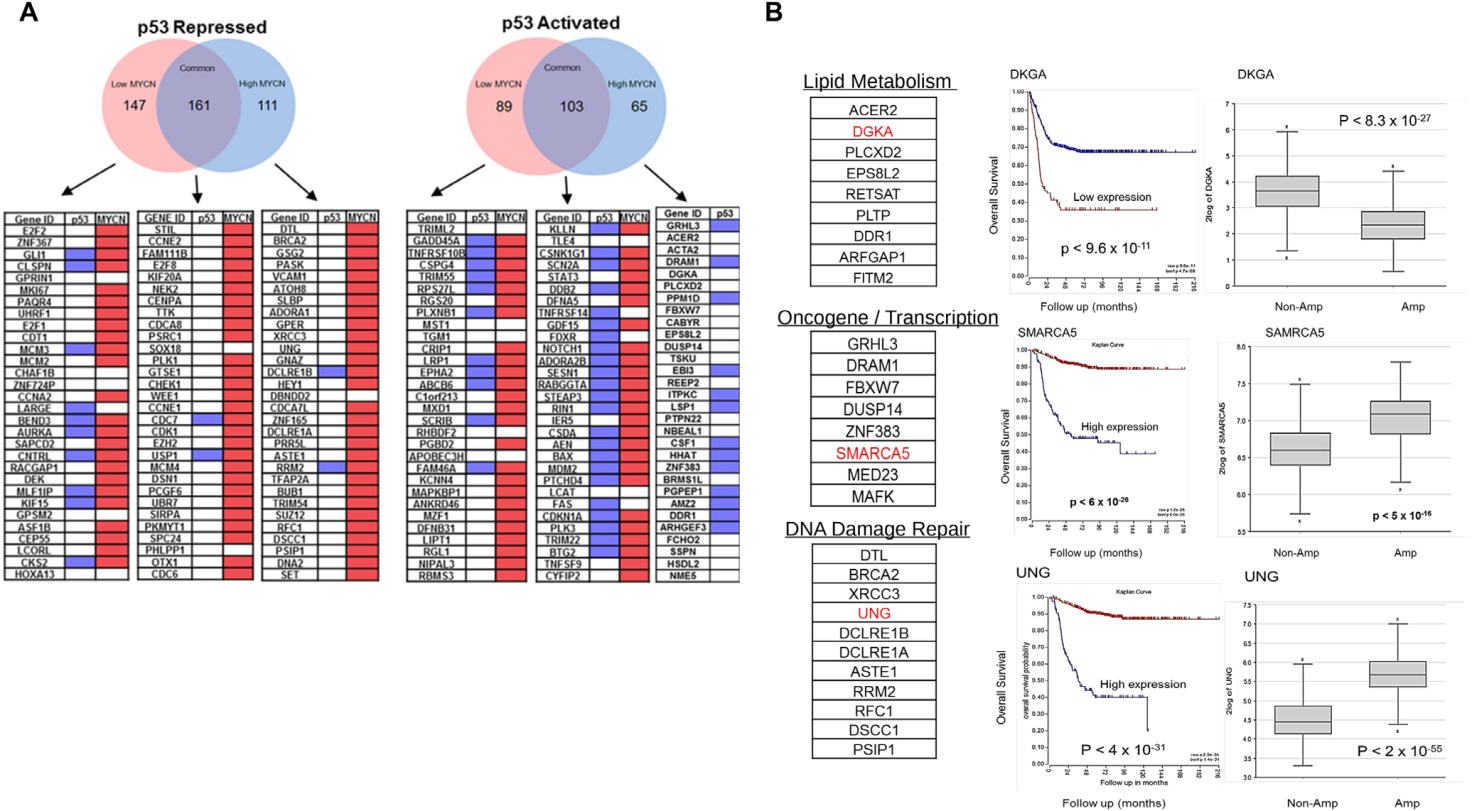
**(A)** The MYCN-ChIP-Seq database and p53-ChIP-Seq composite database were analyzed to determine binding patterns at the MYCN (E-box) and p53 (p53-RE) promoters in the top genes. The p53-repressed and p53-activated genes under low MYCN or high MYCN conditions were analyzed and summarized here for the presence or absence of E-box and p53-REs. Top 30 genes in each category by fold change were analyzed. Red= MYCN E-box present; Blue= p53-RE present. **(B)** Functional annotations of p53 response genes under high MYCN condition were analyzed and found to be highly correlated with worse prognosis and MYCN levels in NB patients. R2: Kosak (n=498) dataset of NB patient was used.

In addition to the modulation of p53 target genes defined in Figure 4, RNA-seq also defined genes regulated by Nutlin-3a treatment only in the presence (or absence) of MYCN induction. These included 176 stress response genes exclusive to the MYCN high condition. A search for promoter E-boxes and p53REs revealed that many of these genes lacked canonical p53 binding sites while almost all had E-boxes (Figure 5A). Gene ontology analysis demonstrated suppression of DNA repair, apoptosis and cell cycle control genes, while metabolic, oncogene signaling, and lipid metabolism genes were increased in response to Nutlin-3a (Figure 5B, Supplementary Figure 3-5). Furthermore, Kaplan Myer analysis of these genes using a large annotated patient cohort revealed that low expression of p53 activated genes and high expression of p53-repressed genes strongly correlated with worse overall survival (Figure 5B, Supplementary Table 2). Interestingly, most of the repressed genes in this group lacked p53 binding sites and only half of the activated genes had typical p53REs in their promoters, supporting a novel transcriptional function for MYCN/p53 complexes.

### MYCN-p53 complex binds to E-Boxes and p53-REs

Based on these findings, we hypothesized that MYCN could directly alter the localization and affinity of p53-chromatin binding. Recently, MYC and MYCN have been shown to broadly alter transcription across the genome and to interact with groups of transcription factors at ‘super-enhancer’ sites [24, 25]. In addition, p53 gain-of-function mutations have been shown to shift p53 binding specificity to alternative DNA sequences [26]. Therefore, we performed composite meta-analyses using MYCN-ChIP-Seq data from the MYCN3 cell line [27] with available MYCN-ChIP-Seq data sets for activating histone marks in other MYCN-amplified neuroblastoma cell lines [25] and available p53-ChIP-Seq data sets [28–32]. This meta-analysis was used to characterize potential binding sites of MYCN and p53 (Figure 5A). As expected, the most enriched motifs in MYCN-ChIP-Seq were the canonical E-box motif for MYCN and MYC as well as the binding motif for MAX (Supplementary Figure 6A). Distribution analysis of genomic elements showed that increased MYCN levels shifted MYCN binding from introns to active promoters (Supplementary Figure 6B). To confirm that MYCN binding increased at regulatory sites of actively transcribed genes, we looked for the presence of H3K27ac marks and RNA polymerase II (Pol II) binding. We also measured the distance of MYCN from transcription start sites (TSS). We observed higher H3K27ac and Pol II sequence-tag density at loci that bind both MYCN and p53 compared with loci that bound p53 alone (Supplementary Figure 7). These meta-analyses support the premise that MYCN co-localizes with p53 at promoters of active p53 target genes. Further, this interaction could repress transcription of a subset of p53 target genes that control proliferation, DNA damage, and cell division.

**Figure 6:**
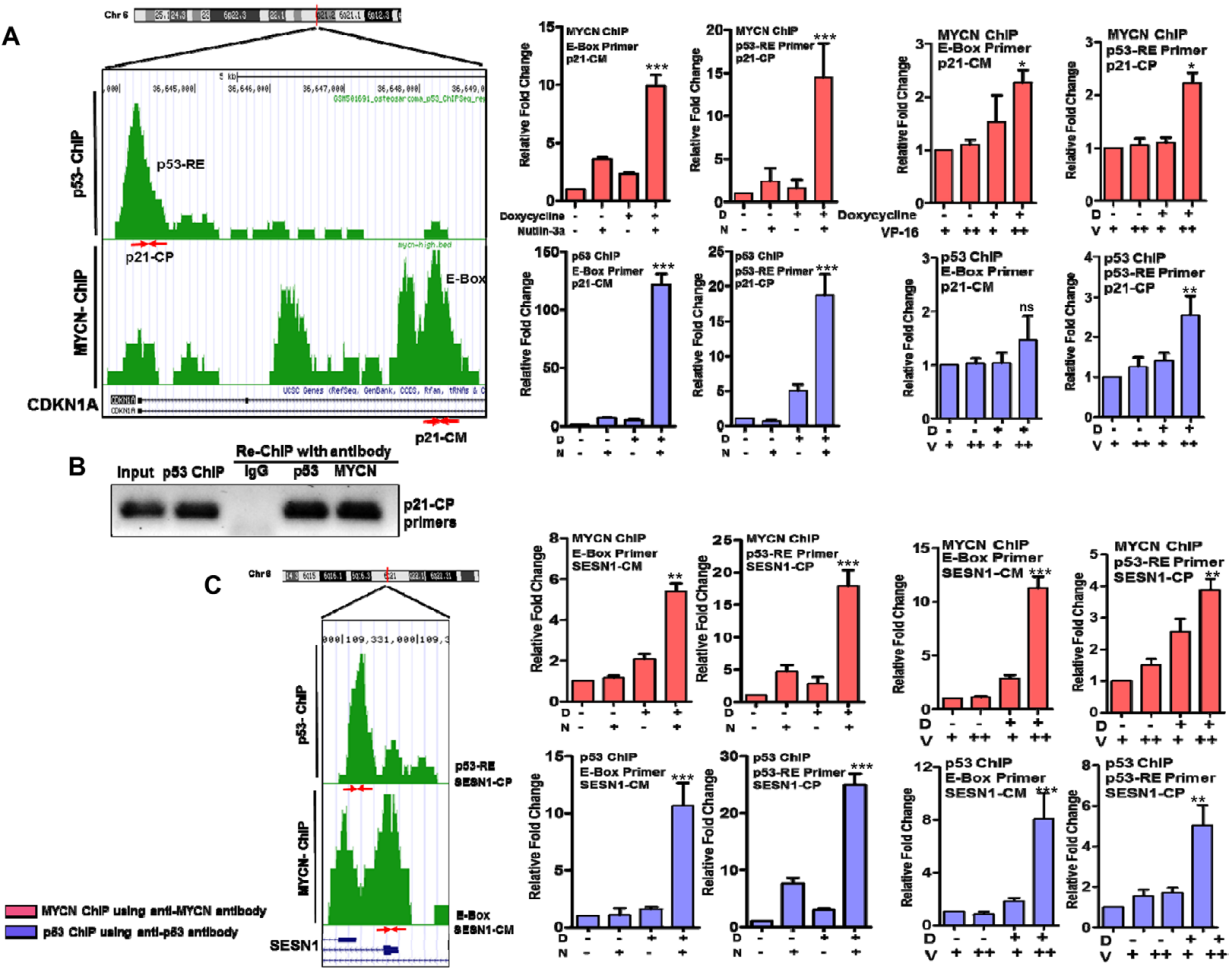

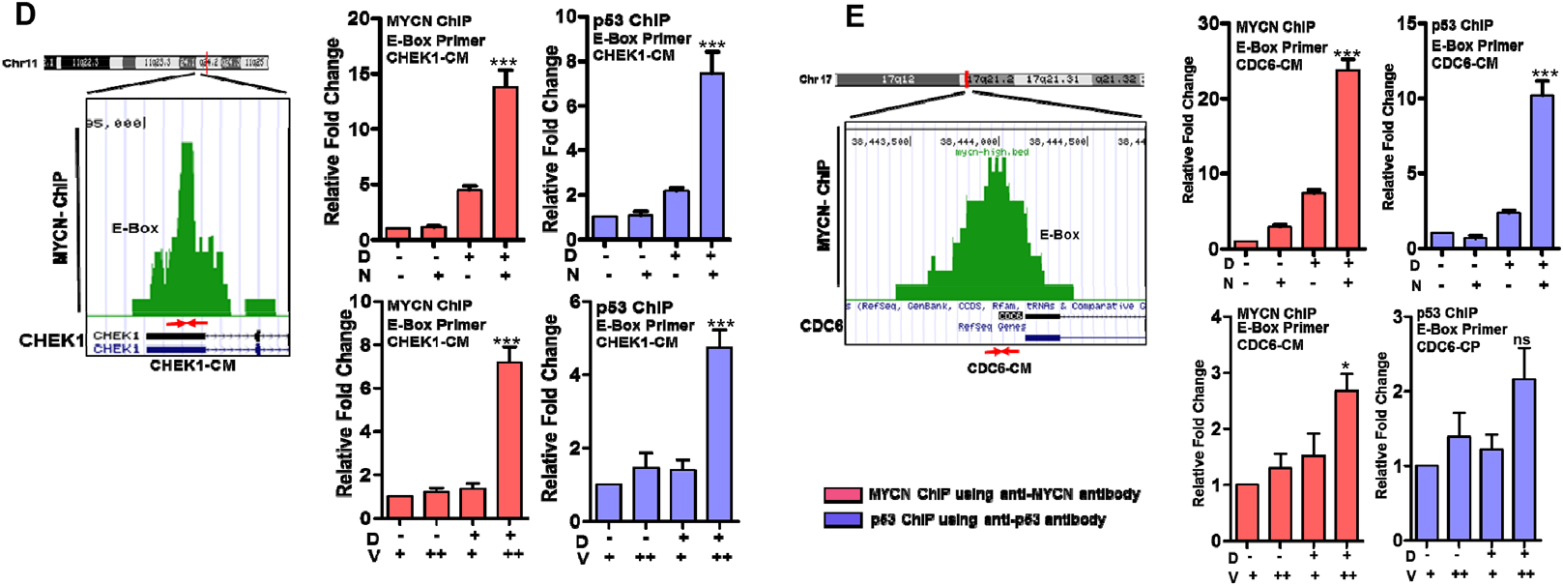
Quantification of p53 and MYCN binding using ChIP-qPCR of p53 and MYCN target genes under different treatment conditions Chip-Seq motif analysis was performed to map p53-RE and MYCN E-Box sites and represented here for individual genes. CDKN1A (p21) and SESN1 are the representative gene selected from the p53-activated group and have both p53-RE and E-box binding sites. CHEK1 and CDC6 are the representative gene selected from the p53-repressed group and have only MYCN E-box binding sites. We designed primers for these mapped sites (listed in Supplementary Table 1) and primer binding locations and respective primer names are shown here with red arrows. MYCN- and p53-ChIP was performed with their respective antibodies as described in Methods. ChIP-qPCR with E-box or p53-RE primers were performed on DNA from both the MYCN-ChIP and p53 ChIP assays, and plotted as individual bar graphs. Cross-ChIP-qPCR experiments using E-box qPCR primers with p53-ChIP DNA or p53-RE primers with MYCN-ChIP DNA were also performed and shown here. The ChIP-qPCR and cross-ChIP-qPCR assays were performed in response to MYCN induction with doxycycline and p53 induction with either Nutlin-3a or with genotoxic chemotherapy treatments. MYCN3 cells were treated with low (10 μg/ml, +) or high (20 μg/ml, ++) doses of VP-16 in the presence or absence of doxycycline for the ChIP assays. D= Doxycycline, N=Nutlin-3a, V=VP-16. **(A)** CDKN1A (p21): primers p21-CP (p53-RE primer) and p21-CM (E-Box primer). ChIP-qPCR and Cross-ChIP-qPCR graphs for p21 locus. **(B)** A Re-ChIP assay was performed for p53 binding site on CDKN1A promoter. The p53 ChIP material was re-ChIPed using either IgG, p53 or MYCN antibody followed and analyzed by PCR amplification using p21-CP primers. The agarose gel is shown with proper Input and loading controls. **(C)** SESN1: primers SESN1-CP (p53-RE primer) and SESN1-CM (E-Box primer). ChIP-qPCR and Cross-ChIP-qPCR graphs for SESN1 locus. **(D)** CHEK1: primers CHEK1-CM (E-Box primer). ChIP-qPCR and ross-ChIP-qPCR graphs for CHEK1 locus. **(E)** CDC6: primers CDC6-CM (E-Box primer). ChIP-qPCR and Cross-ChIP-qPCR graphs for CDC6 locus. ^*^ p<0.05, ^**^ p<0.01, ^***^ p<0.001, ns= non significant.

**Figure 7:**
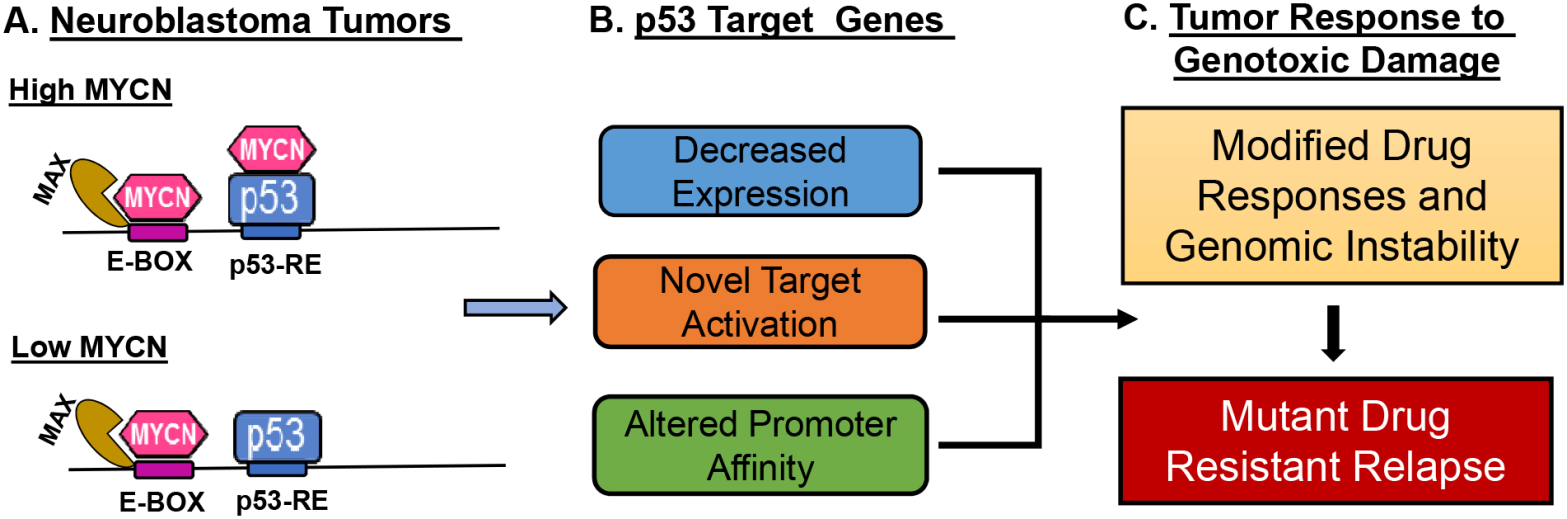
Schematic model for impact of MYCN and p53 interactions on neuroblastoma tumor biology MYCN can complex with p53 via binding to the C-terminal domain when high levels of both MYCN and p53 are present in the nucleus as is the case when p53 wild type/MYCN amplified neuroblastoma is exposed to genotoxic damage. **(A)** Levels of free MYCN alter neuroblastoma responses to therapy **(B)** p53 transcriptional response is modified through changes in chromatin affinity and specificity. **(C)** Changes in DNA damage responses may promote mutation and drug resistance in MYCN amplified cancers.

To quantify changes in MYCN and p53 binding to their cognate response elements when both transcription factors are present at high levels, we next performed direct ChIP-qPCR assays for selected p53 response genes. We designed non-overlapping ChIP-qPCR primers for E-boxes and p53REs (listed in Supplementary Table 1). Typical results are shown for two p53-activated genes, CDKN1a (p21) and SESN1 (Sestrin1) that have both MYCN E-Box and p53-RE binding sites (Figure 6A, 6C); and three p53-repressed genes, CHEK1, CDC6, and CCNE2 that have only E-box and no p53-REs (Figure 6D, 6E and Supplementary Figure 9). We performed standard ChIP-qPCR assays as well as ‘cross ChIP’ (IP with MYCN and qPCR for p53RE and vice versa) in MYCN3 cells with either Doxycycline treatment or not treated to control MYCN levels, and either Nutlin-3a or chemotherapy etoposide (VP-16) treatments to induce p53 levels. Interestingly, we find that binding of both MYCN to E-Boxes and p53 to p53-REs was dramatically enhanced in the presence of high MYCN with high p53, while MAX binding was not significantly increased at well-defined p53 and MYCN targets (Supplementary Figure 8A, 8B). We consistently observed this pattern on all the genes tested in this study (Figure 6). Furthermore, cross-ChIP experiments specifically demonstrated accumulation of p53 associated with E-boxes, and MYCN associated with p53REs for the genes tested. These cross-ChIPs were performed using both anti-p53 and anti-MYCN antibodies to pull down at either E-box motif or p53REs sites. For the CHEK1 and CDC6 genes which lack canonical p53REs and are validated MYCN targets, we could use either anti-p53 or anti-MYCN antibody to pull down at the MYCN binding site by ChIP (Figure 6D, 6E, and Supplementary Figure 9A, 9B). Similar results were observed for p53 induced by the genotoxic chemotherapy VP-16. We confirmed these data using a re-chip assay for p53 at the p21 (CDKN1A) p53RE (Figure 6B). In this assay, we performed p53 ChIP under high-MYCN and Nutlin-3a treatment conditions and re-ChIPed the purified material with either p53 or MYCN antibody and performed ChIP-qPCR with p21 p53RE specific primers. These data support the conclusion that MYCN and p53 can form a complex that associates with both E-Boxes and p53REs and alters transcriptional output. This data also highlights that p53 induced by chemotherapy treatments also form MYCN-p53 complex and alter transcriptional outputs in high-risk neuroblastoma. Overall, our findings support a novel mechanism for MYCN-amplified neuroblastoma where high free MYCN (i.e., not bound to MAX) directly bind to p53 and alter p53 transcriptional responses that may alter the tumor responses to genotoxic chemotherapy (Figure 7).

## DISCUSSION

Amplification of Myc (either MYCN or MYC) is found in a significant proportion of pediatric and adult cancers, including neuroblastoma and subtypes of rhabdomyosarcoma, medulloblastoma, ovarian cancers, lymphomas and lung cancers (http://www.cancerindex.org/geneweb/MYCN.htm#datatable) [33, 34]. Thus, determining how MYCN amplification alters responses to chemotherapy may have broad therapeutic implications. In this study, we used multiple complementary approaches, including RNA-Seq, ChIP-Seq, metadata analysis, and co-IPs, to define a novel direct interaction between MYCN and p53. This interaction alters the transcriptional activation of critical p53 target genes known to regulate DNA-damage repair and apoptotic responses, opposing p53 functions of apoptosis and promoting cell cycle progression and proliferation. Our findings support a role for MYCN modulating genotoxic responses (Figure 7A–7C).

As noted above, MYCN-amplified neuroblastoma has a worse response to therapy and higher rate of both *de novo* drug resistance, treatment associated drug resistance, and recurrence compared to MYCN non-amplified tumors [8, 35]. Myc oncogenes (MYCN and C-Myc) are well known to indirectly oppose p53 by affecting metabolism [36, 37], cell proliferation, and DNA repair [38, 39]. Here we demonstrate p53 activation in the presence of extreme MYCN levels leads to nuclear co-localization of MYCN and p53 and alters transcription of known p53 responsive genes, as well as additional stress responsive genes exclusively regulated in the context of MYCN amplification. Our findings suggest that a direct MYCN-mediated repression/alteration of p53 genotoxic responses may contribute to treatment failure in MYCN-amplified neuroblastoma by further raising the threshold for apoptosis and promoting mutagenic proliferation in the presence of DNA damage. This is consistent with the high mutation rates found in relapsed and progressive neuroblastoma after chemotherapy exposure [40, 41].

We demonstrate MYCN exclusively binds the C-terminal domains of tetrameric p53. Post-translational modifications and the tertiary structure of p53 vitally impact p53 promoter selectivity and downstream transcription [12, 42, 43]. We propose that in addition to modulating p53 promoter selectivity, MYCN binding to the p53 C-terminal domain (CTD) is also likely to interfere with other p53 cofactors (e.g.14-3-3, TAF1 and 53BP1) known to bind this region to modulate p53-mediated stress responses [44]. This interaction may also limit or prevent secondary interactions between p53 and downstream transcription factors, such as DMTF1a and YY-1 [45]. These interactions can both stabilize and promote p53 (DMTF1), or negatively regulate p53 (YY-1) to modulate transcriptional responses to stress [46]. Recently p53 CTD acetylation has been linked to modulation of SET functions essential for p53 activity in normal and stressed cells [14]. Thus, context dependent binding of Myc(N) to the p53 CTD, which is shown to alter acetylation and other PTMs, may have broad impact on p53 responses [47].

Under MYCN non-amplified conditions where MYCN is likely completely associated with its high-affinity binding partner MAX, we find no MYCN associated with p53 in response to DNA damage. Of note MAX levels do not increase with MYCN amplification and are thus limiting in MYCN amplified tumors (Supplementary Figure 8C). This is consistent with recent studies demonstrating a dynamic interaction between p53 and C-Myc regulating transcriptional responses to DNA damage [48]. In this work p53 activation lowered C-Myc levels and this was critical for appropriate p53 dependent apoptosis and cell cycle arrest. In contrast, we demonstrate a pathologic interaction of MYCN with p53 when MYCN is highly over-expressed by amplification.

Importantly, we observe that MYCN amplification alters the repertoire of stress response genes activated and repressed by Nutlin-3a or Etoposide. These include genes with critical roles in DNA damage and proliferation whose expression strongly correlates with poor overall survival (Figure 5). These data are consistent with the recent observations that the p53 CTD critically influences how the p53 DNA binding domain (DBD) binds cognate DNA [12]. Mutations altering post-translational modifications or deletion of the p53 CTD induced internal structural changes in the p53 tetramer significantly altering DNA binding affinity and specificity. Gain-of-function mutations in p53 can also alter DNA binding specificity to generate novel pro-tumorigenic functions [26]. We propose that MYCN or C-Myc binding to the p53 CTD may phenocopy such mutations, altering p53 target selection and limiting transcriptional responses to genotoxic stress. Thus, our proposed alternative mechanisms modulating p53 may play a critical role altering the clinical responses to genotoxic chemotherapy.

## MATERIALS AND METHODS

### Cell culture

The human neuroblastoma cell line SHEP was stably transduced with a vector containing a Tet-inducible human MYCN gene. A stable single clone was selected and named MYCN3. The cells were routinely maintained and cultured as described previously [49]. Cellular p53 levels were increased by treating MYCN3 cells with 5 μM Nutlin-3a for 8 h. MYCN levels were controlled by treating MYCN3 cells with 1 μM doxycycline for 16 h [27]. All cell lines used in the present study were maintained as described previously, checked for MYCN and p53 expression, and validated by genotyping within the past 12 months [50]. All cell lines used were tested for Mycoplasma on a monthly basis.

### Proximity ligation assay (PLA)

MYCN3 cells were plated in 96-well plates with coverslip bottoms that were pre-treated with poly-D-lysine. Cells were treated overnight under the treatment conditions described in the main text. Concentrations of the compound used were as follows: 1 μM doxycycline, 5 μM Nutlin, or media control. Cells were washed and fixed using PHEM buffer. Rabbit anti-MYCN (Santa Cruz Biotechnology) and mouse anti-p53 (DO-1, Santa Cruz Biotechnology) primary antibodies were used to detect the interaction between these two proteins using the Duolink *In Situ* Red kit (Sigma Aldrich) according to the manufacturer’s instructions. Cells were co-stained with DAPI prior to imaging with a Nikon Eclipse Ti microscope. Z-stack images were obtained at 60× magnification, and maximum intensity projections were generated. To quantify PLA spots, size and intensity thresholds were applied to all images, and the number of spots for each cell was scored by attributing the spots to the closest nucleus. Cells with too many spots to count were assigned a value of 125 spots. At least 24 cells were scored per condition.

### Endogenous co-immunoprecipitation assay

Crude nuclear protein extract from Nutlin-3a-treated (10 μM for 4 h) IMR-32 cells and Hela cells were prepared. Nuclei were isolated using hypotonic NP-40 buffer (10 mM HEPES, 50 mM NaCl, 1 mM DTT, 1 mM sodium pyrophosphate, 1 mM sodium orthovanadate, 1 mM sodium fluoride, 1 mM PMSF. cOmplete Protease Inhibitor Cocktail, and 0.2% NP-40) and lysed via sonication in nuclei lysis buffer (50 mM Tris HCl pH 8.00, 150 mM NaCl, 1 mM DTT, 1 mM sodium pyrophosphate, 1 mM sodium orthovanadate, 1 mM sodium fluoride, 1 mM PMSF, cOmplete Protease Inhibitor Cocktail, and 0.05% NP-40). One milligram of crude nuclear protein extract was incubated overnight with 2 μg of anti-p53 antibody (Ab-7, Calbiochem) or 2 μg of control sheep IgG at 4°C. Recombinant Protein G agarose resin (Invitrogen) was used to purify protein complexes. Following three washes with 0.15% NP-40 in nuclei lysis buffer, protein samples were eluted by boiling resin in Laemmli sample buffer, separated using SDS-PAGE, and analyzed using Western blot with an anti-MYCN monoclonal antibody (B8.4.B, Santa Cruz Biotechnology), anti-p53 monoclonal antibody (DO-1, Santa Cruz Biotechnology), anti-MAX antibody (C17, Santa Cruz Biotechnology), and anti-MYC (N262, Santa Cruz Biotechnology).

### Exogenous co-immunoprecipitation assay

Non-small cell lung cancer (p53-/-) were transiently transfected with the following combinations of plasmid constructs: (1) pEGFPN1-p53_GFP/pCMV14 empty, (2) pEGFPN1-p53_GFP/pCMV14- MYCN_3×Flag, and (3) pEGFPN1 empty/pCMV14-MYCN-3×Flag. pEGFPN1-p53_GFP was purchased (#12091; Addgene). Crude nuclear protein fractions were collected as described above. A total of 0.25 mg crude nuclear protein extract was incubated overnight at 4°C with 2 μg of anti-p53 (Ab-7, sheep, Calbiochem) or 2 μg of anti-FLAG antibodies (M2, Sigma Aldrich). Protein complexes were purified, eluted and separated as described above. Western blots were probed using an anti-p53 monoclonal (DO-1, Santa Cruz Biotechnology) and anti-FLAG antibodies (M2, Sigma Aldrich).

### GST pull-down assay to determine p53 and MYCN direct binding

Full-length coding sequences of p53 and MYCN were cloned into the pGEX-2T (N-terminal GST tag) and pET-22b plasmid (C-terminal 6×His tag), respectively. Primer sequences are listed in Supplementary Table 1. BL21 *E. coli* were transformed with either MYCN-6×His or GST-p53 vector and protein expression was induced with 0.4 mM IPTG for 8 h at 25°C. GST-p53 cells were lysed in GST lysis buffer (1% Triton, 1 μg/μl lysozyme, 0.5 mM EDTA, and 1 mM PMSF in phosphate buffered saline), purified and immobilized on glutathione-agarose beads (Sigma Aldrich). BL-21 *E.Coli* cells expressing MYCN-6×His were lysed in His lysis buffer (50 mM NaH2PO4, 1% Triton, 1 μg/μl lysozyme, 1 mM PMSF, 300 mM NaCl, and 20 mM imidazole), purified using HIS-Select Nickel Affinity Gel (Sigma Aldrich) and eluted with 350 mM imidazole in His lysis buffer without lysozyme. Purified MYCN-6×His protein was incubated with glutathione beads coated with equimolar amounts of either purified GST or GST-p53 overnight at 4°C. Pull-down complexes were eluted by boiling samples in Laemli sample buffer, separated using SDS-PAGE, and analyzed using Western blot with anti-MYCN monoclonal antibody (B8.4.B, Santa Cruz Biotechnology) and Stain-Free total protein staining was detected via UV activation. Data were collected using a ChemiDoc MP (Bio- Rad). Full-length p53-GST construct was used to generate the p53 binding mutants. The GST-p53 L344A can only form the p53 dimeric form and GST-p53 L344P can only form monomeric form, while the GST-p53 WT can form p53 tetramers. All of these constructs were analyzed as described above by immunoprecipitation for GST followed by Western blotting for MYCN.

### GST pull-down assay to determine p53 and C-Myc binding

The C-Myc coding sequence was cloned into a pGEX-2T plasmid in frame with the N-terminal GST. Primer sequences are provided in Supplementary Table 1. Cloned vectors were transformed into BL21 E. Coli, and protein expression was induced with 0.4 mM IPTG at 37°C for 4h. Cells were lysed in GST lysis buffer. Recombinant proteins were purified and immobilized onto glutathione-agarose beads as described above. Immobilized GST-C-Myc fragments were incubated overnight at 4°C with 50 μg of HEK-293T nuclear extracts transiently over-expressing full-length p53 protein. Protein complexes were purified, eluted, and separated as described above. Western blots were analyzed using anti-p53 monoclonal (DO-1) antibody.

### GST pull-down assay to map p53-MYCN binding

Different mutated forms or truncations of p53 and MYCN were cloned into pGEX-2T plasmids in frame with the N-terminal GST. Primer sequences are listed in Supplementary Table 1. pGEX2T p53 and MYCN vectors were transformed into BL21 *E. Coli* strain and expression was induced with 0.4 mM IPTG for 8 h at 25°C. Recombinant proteins were purified and immobilized as described above. Immobilized GST-p53 fragments were incubated overnight at 4°C with 50 μg of SK-N-BE 2 (c) nuclear extracts. GST-MYCN fragments were incubated overnight at 4°C with 50 μg of HEK-293T (transiently over-expressing full-length p53 protein) nuclear extracts. Protein complexes were purified, eluted and separated as described above. Western blots were analyzed using anti-p53 (DO-1), anti-MYCN monoclonal (B8.4.B) and anti-MAX antibody (C17).

### RNA sequencing analysis

Total RNA was isolated and RNA-Seq was performed using the HiSeq™ platform (Illumina). Approximately 40–77 million read pairs was observed for each sample. Reads were mapped onto the human genome build UCSC hg19 (NCBI 37) and the GENCODE human gene model using TopHat2. Gene expression was calculated using Cufflinks2 platform. The profiles of all samples were combined, and quantile normalization was applied to determine the final fragments per kilobase of transcript per million mapped reads (FPKM) values. Analysis of differentially expressed genes was performed using t-test statistics in MeV statistical software. Significance was defined as log (fold changes)>1.3, *p*<0.05, and FPKM>1. Significantly enriched pathways and processes were determined using multiple methods. First, significant genes were analyzed using DAVID platform (https://david.ncifcrf.gov/) [51]. Next, we evaluated enriched pathways using gene set enrichment analysis (GSEA), and the pathway compendium was compiled using the Molecular Signature Database (MSigDB) to screen for pathways and processes. Principal component analysis, hierarchical clustering, and heatmaps were generated using the numpy and scipy scientific Python packages.

### ChIP-seq analysis

We used previously reported MYCN chromatin immunoprecipitation and sequencing (ChIP-Seq) datasets from neuroblastoma cells with high and low MYCN levels [27]. Each library yielded 5–7 million sequenced reads that were mapped using Burrows-Wheeler alignment to human genome build UCSC hg19/NCBI. Genome-wide maps were visualized using the Integrative Genomics Viewer or the UCSC Genome Browser (http://genome.ucsc.edu/). MACS2 software was used to identify enriched regions. To determine potential genome-wide p53-binding sites, we downloaded publicly available p53-ChIP-Seq datasets from multiple cell types [28–32]. We determine MYCN peaks that overlapped with potential p53-binding sites using the BEDTOOLS software. Selected gene targets were analyzed using GSEA as described above.

### Chromatin immunoprecipitation-qPCR

ChIP was performed on 1×10^7^ MYCN3 neuroblastoma cells using the ChIP-IT Express Chromatin Immunoprecipitation Kit (Active Motif) according to the manufacturer’s instructions. Samples were cultured in one of four conditions: condition A, DMSO; condition B, Nutlin-3a; condition C, doxycycline; and condition D, doxycycline and Nutlin-3a. In another experiment to determine effect of genotoxic chemotherapy, cells were treated with 10 μg/ml or 20 μg/ml of etoposide (VP-16) in the presence or absence of doxycycline. Samples were sonicated for 20 cycles in 30 sec intervals using a Bioruptor UCD-200 sonicator (Diagenode). ChIP was performed using negative control antibody (IgG control, 12-370, EMD Millipore), anti-human p53 antibody (EpiTect ChIP-Grade Antibody Kit (p53), GAH-112, EMD Millipore), and ChIP-grade Anti-MYCN antibody (ab16898, Abcam). Input was generated by purifying DNA from sonicated lysates of each sample. Different gene promoters were analyzed for MYCN and p53 binding by analyzing the MYCN-ChIP-sequencing dataset [27] and publicly available p53-ChIP-Seq datasets. ChIP-qPCR primers were designed for MYCN- and p53-binding sites on promoters (listed in Supplementary Table 1). Real-time PCR reactions were performed in triplicate as described previously [52]. Dissociation curves were analyzed for each primer pair to ensure amplicon quality and monitor for primer dimers. Input and negative control IgG-ChIP samples were also analyzed for each sample. The amount of genomic DNA that co-precipitated with a specific antibody was calculated by comparing it to total input DNA used for each immunoprecipitation. Fold enrichment above background was calculated by normalizing against control IgG-ChIP. The qPCR reactions were performed in triplicate for each sample, input, and control IgG.

### ChIP and Re-ChIP assay in IMR-32 neuroblastoma cell line

IMR-32 cells (1×10^7^ cells) were cross-linked using 1% formaldehyde, and the reaction was stopped using 0.125 M glycine. The cell pellet was resuspended in cell lysis buffer, sonicated, and centrifuged at 6000 rpm. Then, RIPA buffer was added to lyse nuclei. DNA was sheared by sonicating with Bioruptor (Diagenode). A small aliquot of sonicated material was set aside, and remaining sample was immunoprecipitated with 5 μg of ChIP-grade antibodies. Rec-sepharose Protein A or G beads (Invitrogen) were used to immobilize immuno-complexes. Following an RNAse A treatment at 37°C for 1 h, cross-linking was reversed using Proteinase K (Roche) for 6 hours at 65°C. Immunoprecipitated DNA was purified using phenol/chloroform and ethanol precipitation techniques. DNA-protein complexes were eluted by adding elution buffer (DTT 15mM, SDS 2%, Complete inhibitor and PMSF 1mM) at 37°C 2h on shaking. Eluted samples were diluted (20-fold) in RIPA buffer without SDS and immunoprecipitated with 5 μg of ChIP grade anti-MYCN, anti p53 and control IgG antibodies. Rec-sepharose Protein A or G beads (Invitrogen) were used to immobilize immuno-complexes. Following an RNAse A treatment at 37°C for 1 h, cross-linking was reversed using Proteinase K (Roche) for 6 hours at 65°C. Immunoprecipitated DNA was purified using phenol/chloroform and ethanol precipitation techniques. Antibodies used in this study were as follows: anti-MYCN (B8.4.B), anti-MAX (C-17), and anti-p53 (DO-1). DNA was analyzed using qPCR. Primer sequences are presented in Supplementary Table 1.

### Clinical patient cohorts

Neuroblastoma patient dataset of Kocak (N=649) that include microarray profiles of unique primary tumors are publically available from the R2: Genomic Analysis and Visualization Platform database (http://hgserver1.amc.nl/cgibin/r2/main.cgi). This dataset was analyzed for overall survival correlation with gene expression and for correlation between genes, as described previously [50].

### Statistical analysis

Data values are expressed as mean ± SEM unless otherwise stated as S.D for standard deviation. Data values were compared using two-tailed Mann-Whitney test for non-normally distributed variables. Student’s t-test was used for normally distributed variables with one-tailed tests used for data with unequal variance and two-tailed tests used for all other analyses. All the experiments were repeated at least three times and all measurements were made in triplicates.

### Databases

The RNA-Seq and ChIP-Seq data have been deposited in the Gene Expression Omnibus repository. The accession number for the RNA-Seq data is GSE83328 and for the ChIP-Seq data is GSE83317.

## SUPPLEMENTARY MATERIALS FIGURES AND TABLES




