# Correction: MYCN acts as a direct co-regulator of p53 in MYCN amplified neuroblastoma

**DOI:** 10.18632/oncotarget.25776

**Published:** 2018-07-06

**Authors:** Saurabh Agarwal, Giorgio Milazzo, Kimal Rajapakshe, Ronald Bernardi, Zaowen Chen, Barbieri Eveline, Jan Koster, Giovanni Perini, Cristian Coarfa, Jason M. Shohet

**Affiliations:** ^1^ Division of Hematology-Oncology, Department of Pediatrics, Baylor College of Medicine, Houston, Texas, USA; ^2^ Department of Pharmacy and Biotechnology, University of Bologna, Bologna, Italy; ^3^ Dan L Duncan Cancer Center, Department of Molecular and Cellular Biology, Baylor College of Medicine, Houston, Texas, USA; ^4^ Department of Oncogenomics, Academic Medical Center, University of Amsterdam, Amsterdam, The Netherlands

**This article has been corrected:** The corrected author name is given below:

**Barbieri Eveline**

Original article: Oncotarget. 2018; 9:20323-20338. https://doi.org/10.18632/oncotarget.24859

