# Supplementary material for: MYCN acts as a direct co-regulator of p53 in MYCN amplified neuroblastoma
Source: Oncotarget. 2018 Apr 17;9(29):20323–38. doi: 10.18632/oncotarget.24859 (PMC5945521; doi:10.18632/oncotarget.24859)
Supplement: Supplementary file 1 [file oncotarget-09-20323-s001.pdf]

MYCN acts as a direct co-regulator of p53 in MYCN amplified neuroblastoma

SUPPLEMENTARY MATERIALS

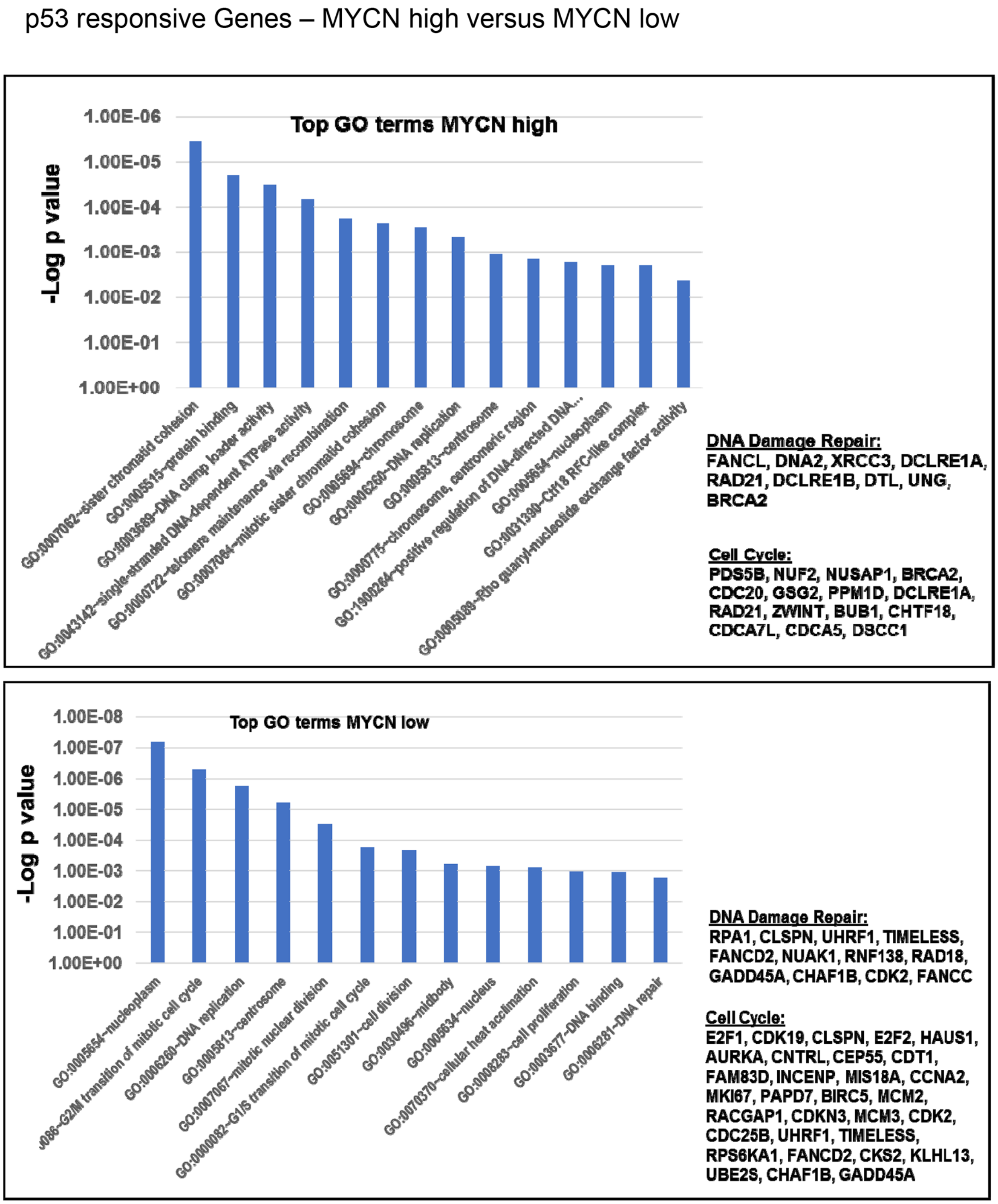

Supplementary Figure 1: Gene ontology analysis of p53 responsive genes comparing MYCN high versus MYCN Low groups to determine their functional grouping using the DAVID bioinformatics platform (https://david.ncifcrf.gov/).

Functional annotations for p53 targets modulated by MYCN

Functional annotation Term	PValue	Bonferroni	FDR
GO:0007049~cell cycle	3.57E-18	7.99E-16	4.56E-15
GO:0000278~mitotic cell cycle	2.96E-13	6.62E-11	3.77E-10
GO:0005819~spindle	4.15E-13	2.28E-11	4.08E-10
GO:0015630~microtubule cytoskeleton	3.22E-12	1.77E-10	3.17E-09
GO:0022403~cell cycle phase	3.37E-11	7.55E-09	4.31E-08
GO:0022402~cell cycle process	4.29E-11	9.61E-09	5.48E-08
GO:0000279~M phase	9.16E-11	2.05E-08	1.17E-07
GO:0044430~cytoskeletal part	1.96E-09	1.08E-07	1.93E-06
GO:0007067~mitosis	2.53E-09	5.67E-07	3.23E-06
GO:0000280~nuclear division	2.53E-09	5.67E-07	3.23E-06
GO:0000087~M phase of mitotic cell cycle	2.92E-09	6.54E-07	3.73E-06
GO:0048285~organelle fission	3.48E-09	7.78E-07	4.44E-06
GO:0051301~cell division	2.53E-08	5.66E-06	3.22E-05
GO:0005856~cytoskeleton	1.33E-07	7.31E-06	1.31E-04
GO:0007017~microtubule-based process	1.94E-07	4.35E-05	2.48E-04
GO:0043232~intracellular non-membrane-bounded org	2.17E-07	1.19E-05	2.13E-04
GO:0043228~non-membrane-bounded organelle	2.17E-07	1.19E-05	2.13E-04

	Cell cycle checkpoint
ID	Gene Name
BRIP1	BRCA1 interacting protein C-terminal helicase 1
CHEK1	CHK1 checkpoint homolog (S. pombe)
GTSE1	G-2 and S-phase expressed 1
TTK	TTK protein kinase
CDK1	cell division cycle 2, G1 to S and G2 to M
CENPF	centromere protein F, 350/400ka (mitosin)
CCNE2	cyclin E2

	Kinetochore
ID	Gene Name
NDC80	NDC80 homolog, kinetochore complex component
NEK2	NIMA (never in mitosis gene a)-related kinase 2
CENPA	centromere protein A
CENPF	centromere protein F, 350/400ka (mitosin)
SKA1	chromosome 18 open reading frame 24
KIF2C	kinesin family member 2C
PLK1	polo-like kinase 1 (Drosophila)

	Protein Kinases
ID	Gene Name
CHEK1	CHK1 checkpoint homolog (S. pombe)
NEK2	NIMA (never in mitosis gene a)-related kinase 2
TTK	TTK protein kinase
WEE1	WEE1 homolog (S. pombe)
CDK1	cell division cycle 2, G1 to S and G2 to M
CDC7	cell division cycle 7 homolog (S. cerevisiae)
PLK1	polo-like kinase 1 (Drosophila)

	Cell Division
ID	Gene Name
NDC80	NDC80 homolog, kinetochore complex component (S. cerevisia
NEK2	NIMA (never in mitosis gene a)-related kinase 2
WEE1	WEE1 homolog (S. pombe)
CDK1	cell division cycle 2, G1 to S and G2 to M
CDC25A	cell division cycle 25 homolog A (S. pombe)
CDC7	cell division cycle 7 homolog (S. cerevisiae)
CDCA2	cell division cycle associated 2
CDCA8	cell division cycle associated 8
CENPF	centromere protein F, 350/400ka (mitosin)
SKA1	chromosome 18 open reading frame 24
CCNB1	cyclin B1
CCNB2	cyclin B2
CCNE1	cyclin E1
CCNE2	cyclin E2
KIF11	kinesin family member 11
KIF23	kinesin family member 23
KIFC1	kinesin family member C1
NCAPG	non-SMC condensin I complex, subunit G
PLK1	polo-like kinase 1 (Drosophila)

Supplementary Figure 2: Details of functional annotations of p53 target genes regulation by p53 activation is dependent on MYCN status, highlighting cell cycle checkpoint, cell division and replication associated genes.

p53 activated genes exclusive to MYCN high context

Functional annotation Term	PValue	Bonferroni	FDR
GO:0007049~cell cycle	3.57E-18	7.99E-16	4.56E-15
GO:0000278~mitotic cell cycle	2.96E-13	6.62E-11	3.77E-10
GO:0005819~spindle	4.15E-13	2.28E-11	4.08E-10
GO:0015630~microtubule cytoskeleton	3.22E-12	1.77E-10	3.17E-09
GO:0022403~cell cycle phase	3.37E-11	7.55E-09	4.31E-08
GO:0022402~cell cycle process	4.29E-11	9.61E-09	5.48E-08
GO:0000279~M phase	9.16E-11	2.05E-08	1.17E-07
GO:0044430~cytoskeletal part	1.96E-09	1.08E-07	1.93E-06
GO:0007067~mitosis	2.53E-09	5.67E-07	3.23E-06
GO:0000280~nuclear division	2.53E-09	5.67E-07	3.23E-06
GO:0000087~M phase of mitotic cell cycle	2.92E-09	6.54E-07	3.73E-06
GO:0048285~organelle fission	3.48E-09	7.78E-07	4.44E-06
GO:0051301~cell division	2.53E-08	5.66E-06	3.22E-05
GO:0005856~cytoskeleton	1.33E-07	7.31E-06	1.31E-04
GO:0007017~microtubule-based process	1.94E-07	4.35E-05	2.48E-04
GO:0043232~intracellular non-membrane-bounded org	2.17E-07	1.19E-05	2.13E-04
GO:0043228~non-membrane-bounded organelle	2.17E-07	1.19E-05	2.13E-04

	Cell cycle checkpoint
ID	Gene Name
BRIP1	BRCA1 interacting protein C-terminal helicase 1
CHEK1	CHK1 checkpoint homolog (S. pombe)
GTSE1	G-2 and S-phase expressed 1
TTK	TTK protein kinase
CDK1	cell division cycle 2, G1 to S and G2 to M
CENPF	centromere protein F, 350/400ka (mitosin)
CCNE2	cyclin E2

	Kinetochore
ID	Gene Name
NDC80	NDC80 homolog, kinetochore complex component
NEK2	NIMA (never in mitosis gene a)-related kinase 2
CENPA	centromere protein A
CENPF	centromere protein F, 350/400ka (mitosin)
SKA1	chromosome 18 open reading frame 24
KIF2C	kinesin family member 2C
PLK1	polo-like kinase 1 (Drosophila)

	Cell Division
ID	Gene Name
NDC80	NDC80 homolog, kinetochore complex component (S. cerevisi
NEK2	NIMA (never in mitosis gene a)-related kinase 2
WEE1	WEE1 homolog (S. pombe)
CDK1	cell division cycle 2, G1 to S and G2 to M
CDC25A	cell division cycle 25 homolog A (S. pombe)
CDC7	cell division cycle 7 homolog (S. cerevisiae)
CDCA2	cell division cycle associated 2
CDCA8	cell division cycle associated 8
CENPF	centromere protein F, 350/400ka (mitosin)
SKA1	chromosome 18 open reading frame 24
CCNB1	cyclin B1
CCNB2	cyclin B2
CCNE1	cyclin E1
CCNE2	cyclin E2
KIF11	kinesin family member 11
KIF23	kinesin family member 23
KIFC1	kinesin family member C1
NCAPG	non-SMC condensin I complex, subunit G
PLK1	polo-like kinase 1 (Drosophila)

	Lipid Metabolism
ID	Gene Name
ARFGAP1	ADP ribosylation factor GTPase activating protein 1
EPS8L2	EPS8 like 2
ACER2	alkaline ceramidase 2
DGKA	diacylglycerol kinase alpha
DDR1	discoidin domain receptor tyrosine kinase 1
FITM2	fat storage inducing transmembrane protein 2
PLTP	phospholipid transfer protein

	Protein Kinases
ID	Gene Name
CHEK1	CHK1 checkpoint homolog (S. pombe)
NEK2	NIMA (never in mitosis gene a)-related kinase 2
TTK	TTK protein kinase
WEE1	WEE1 homolog (S. pombe)
CDK1	cell division cycle 2, G1 to S and G2 to M
CDC7	cell division cycle 7 homolog (S. cerevisiae)
PLK1	polo-like kinase 1 (Drosophila)

Supplementary Figure 3: Transcriptional analysis by RNA-seq reveals genes modulated by p53 activation exclusively in the presence of MYCN. Show is gene ontology and top enriched classes of genes that activated by p53 activation solely in the context of high MYCN levels.

p53 repressed genes – exclusive to MYCN high condition.

Term	PValue	Bonferroni	FDR
GO:0006259~DNA metabolic process	5.88E-09	5.18E-06	9.14E-06
GO:0051301~cell division	1.93E-08	1.70E-05	3.00E-05
GO:0005694~chromosome	1.58E-07	2.64E-05	1.92E-04
GO:0007059~chromosome segregation	5.16E-07	4.54E-04	8.02E-04
GO:0000280~nuclear division	7.23E-07	6.36E-04	1.12E-03
GO:0007067~mitosis	7.23E-07	6.36E-04	1.12E-03
GO:0000087~M phase of mitotic cell cycle	8.53E-07	7.50E-04	1.33E-03
GO:0006281~DNA repair	9.47E-07	8.33E-04	1.47E-03
GO:0048285~organelle fission	1.04E-06	9.17E-04	1.62E-03
GO:0006974~response to DNA damage stimulus	2.06E-06	1.81E-03	3.20E-03
GO:0043232~intracellular non-membrane-bounded	3.89E-06	6.50E-04	4.74E-03
GO:0043228~non-membrane-bounded organelle	3.89E-06	6.50E-04	4.74E-03
GO:0000279~M phase	3.95E-06	3.47E-03	6.15E-03
GO:0022403~cell cycle phase	6.03E-06	5.29E-03	9.37E-03
GO:0044427~chromosomal part	1.10E-05	1.83E-03	1.34E-02
GO:0000278~mitotic cell cycle	1.20E-05	1.05E-02	1.87E-02
GO:0006260~DNA replication	1.75E-05	1.53E-02	2.72E-02
GO:0022402~cell cycle process	2.83E-05	2.46E-02	4.39E-02

	Cell Cycle
ID	Gene Name
DCLRE1A	DNA cross-link repair 1A (PSO2 homolog, S. cerevisiae)
KIAA1009	KIAA1009
NUF2	NUF2, NDC80 kinetochore complex component, homolog
PDS5B	PDS5, regulator of cohesion maintenance, homolog B
rad21	RAD21 homolog (S. pombe)
ZWINT	ZW10 interactor
BRCA2	breast cancer 2, early onset
BUB1	budding uninhibited by benzimidazoles 1 homolog (yeast)
CDC20	cell division cycle 20 homolog (S. cerevisiae)
CDCA5	cell division cycle associated 5
DSCC1	defective in sister chromatid cohesion 1 homolog (S. cerevisiae)
ID4	inhibitor of DNA binding 4, dominant negative helix-loop-helix
NUSAP1	nucleolar and spindle associated protein 1

	Mitosis
ID	Gene Name
DCLRE1A	DNA cross-link repair 1A (PSO2 homolog, S. cerevisiae)
KIAA1009	KIAA1009
NUF2	NUF2, NDC80 kinetochore complex component, homolog
PDS5B	PDS5, regulator of cohesion maintenance, homolog B
rad21	RAD21 homolog (S. pombe)
ZWINT	ZW10 interactor
BUB1	budding uninhibited by benzimidazoles 1 homolog (yeast)
CDC20	cell division cycle 20 homolog (S. cerevisiae)
CDCA5	cell division cycle associated 5
NUSAP1	nucleolar and spindle associated protein 1

	DNA Repair
ID	Gene Name
DCLRE1A	DNA cross-link repair 1A (PSO2 homolog, S. cerevisiae)
DCLRE1B	DNA cross-link repair 1B (PSO2 homolog, S. cerevisiae)
DNA2	DNA replication helicase 2 homolog (yeast)
FANCL	Fanconi anemia, complementation group L
rad21	RAD21 homolog (S. pombe)
XRCC3	X-ray repair complementing defective repair
ASTE1	asteroid homolog 1 (Drosophila)
BRCA2	breast cancer 2, early onset
C19orf40	chromosome 19 open reading frame 40
RFC1	replication factor C (activator 1) 1, 145kDa
RFC5	replication factor C (activator 1) 5, 36.5kDa
UNG	uracil-DNA glycosylase

Supplementary Figure 4: Gene ontology and top enriched classes of genes repressed by p53 activation solely in the context of high MYCN levels.

KEGG analysis:
Cell cycle repression by p53 in presences or absence of MYCN induction

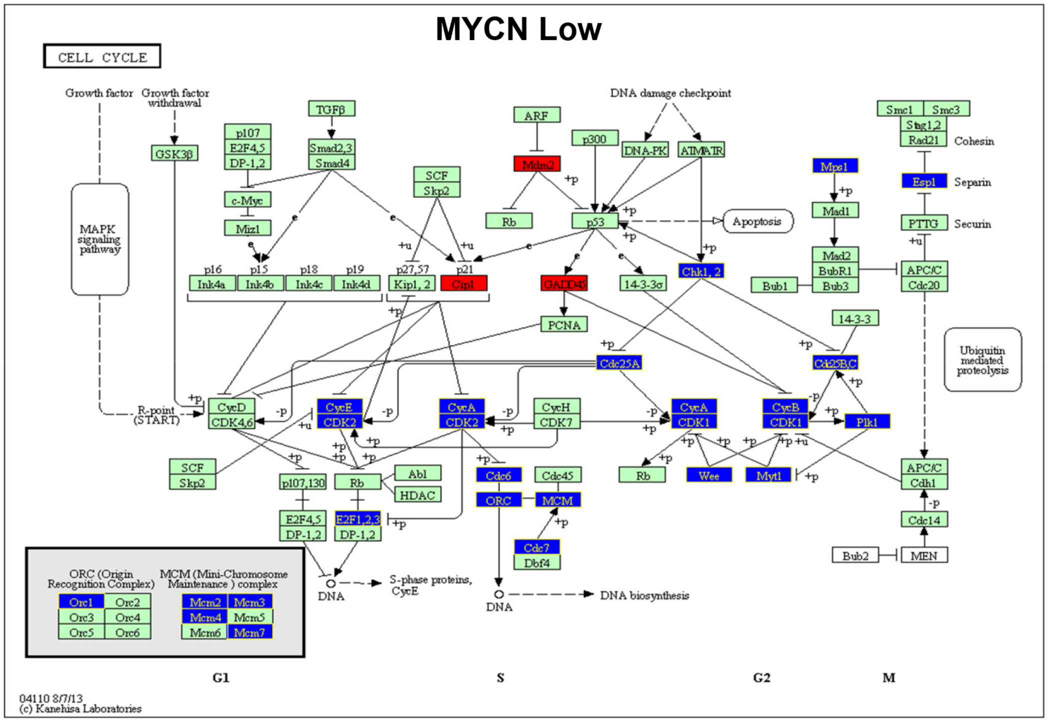

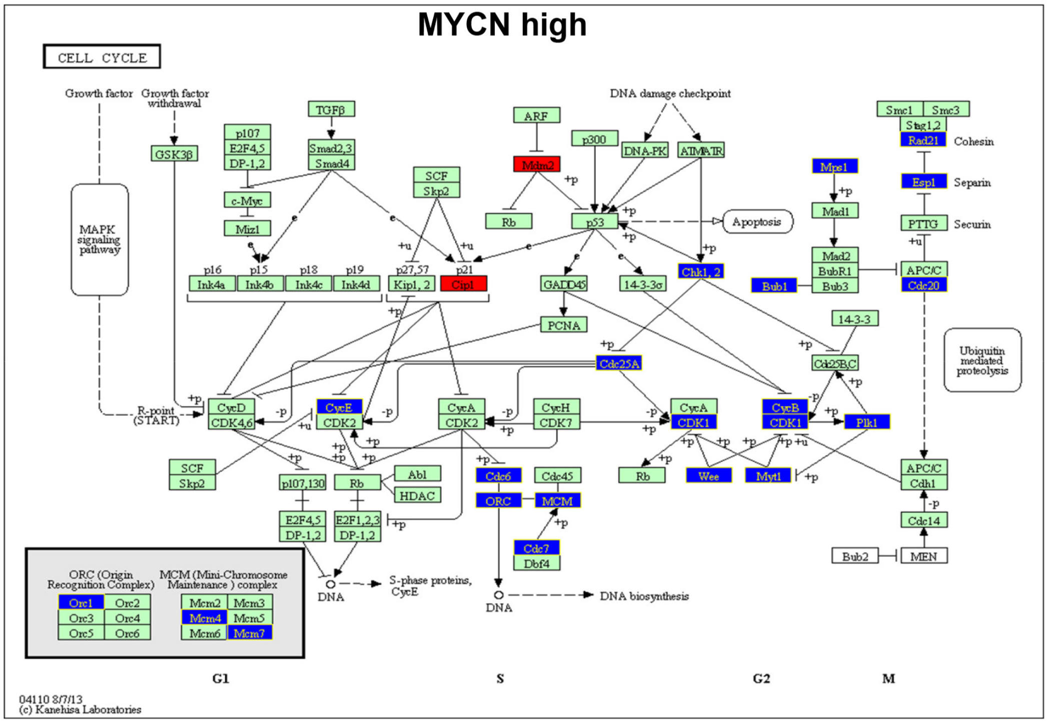

Supplementary Figure 5: Over view of KEGG analysis cell cycle pathways in response to p53 activation with Nutlin. Blue boxes are downregulated genes and red boxes are upregulated genes with and without MYCN induction.

A

Motif	MYCN-Low	MYCN-High	Motif Sequence
MYCN	10^{-559}	10^{-552}	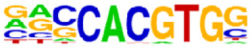
MAX	10^{-527}	10^{-515}	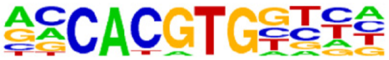
c-MYC	10^{-416}	10^{-407}	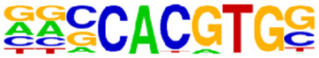

B

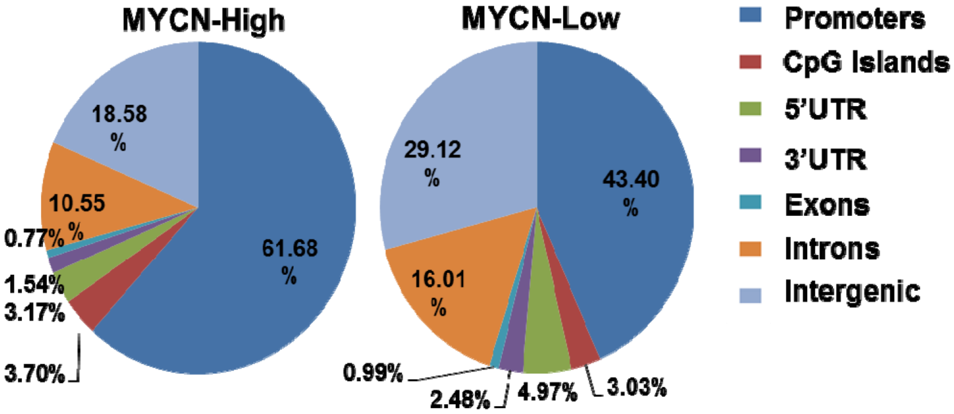

Supplementary Figure 6: (A) Motif analysis of the MYCN-ChIP-Seq data set showed that MYC and MAX were enriched at canonical E-boxes. **(B)** Distribution analysis of MYCN binding over genomic and epigenomic landmarks indicates increased binding at promoters and decreased binding at introns and intergenic regions in high MYCN conditions.

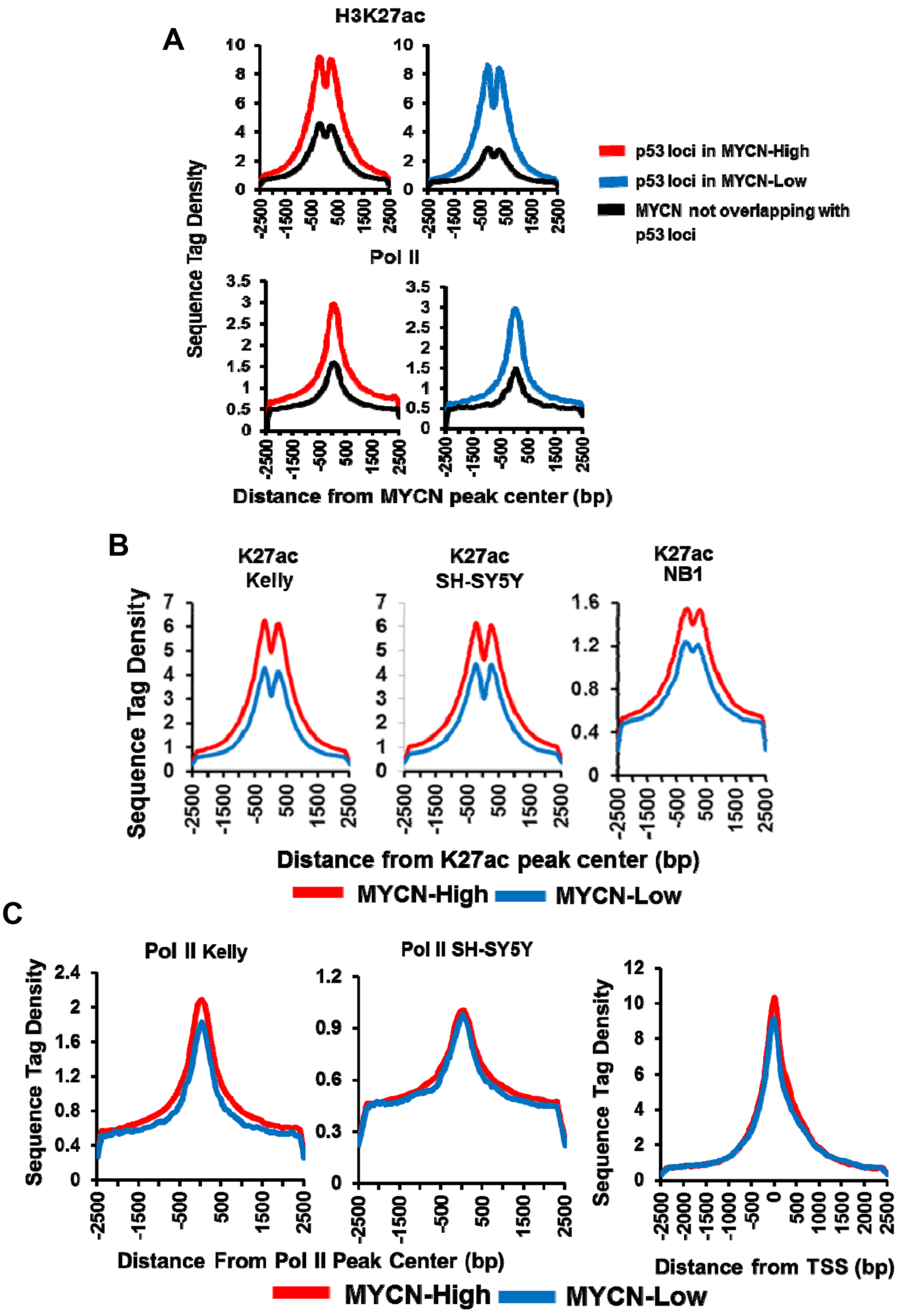

Supplementary Figure 7: (A) Meta-analysis of MYCN-ChIP-Seq datasets suggest a direct interaction between MYCN and p53 at actively transcribed genes. When at high levels, MYCN is found more frequently at transcription regulation sites, as indicated by H3K27ac binding. Meta-analysis of p53-ChIP-Seq binding data sets and MYCN-ChIP-Seq data showed that MYCN peaks overlap with p53-bound loci and are characterized by increased H3K27ac and Pol II. This observation suggests a direct interaction between MYCN and p53 at genes modulated by both transcriptional regulators. **(B)** High levels of MYCN were detected at transcription regulation sites, as indicated by H3K27ac binding. Datasets from the neuroblastoma cell lines Kelly, SH-SY5Y, and NB1 were analyzed. **(C)** Meta-analysis of MYCN-ChIP-Seq data for p53 sites with Pol II enrichment in the SH-SY5Y (MYCN-non-amplified) and Kelly (MYCN-amplified) neuroblastoma cell lines. Localization of MYCN binding peaks near TSS start sites of p53 target genes.

MYCN levels do not alter MAX chromatin binding

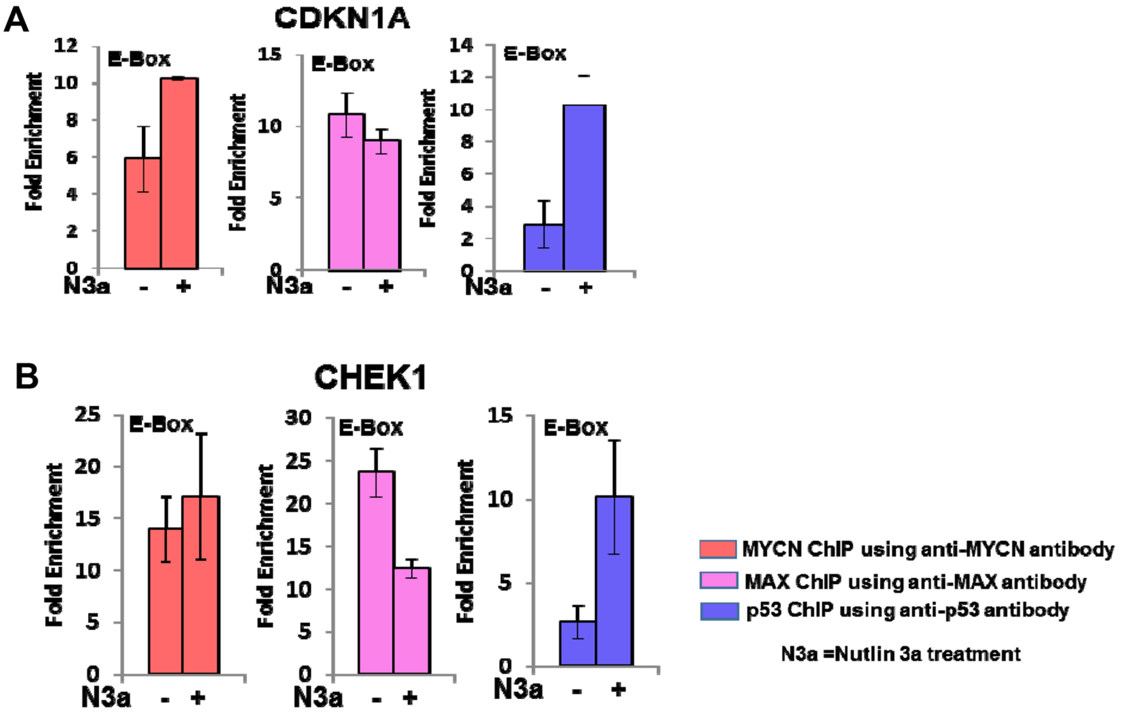

MAX levels do not vary with MYCN levels in Neuroblastoma

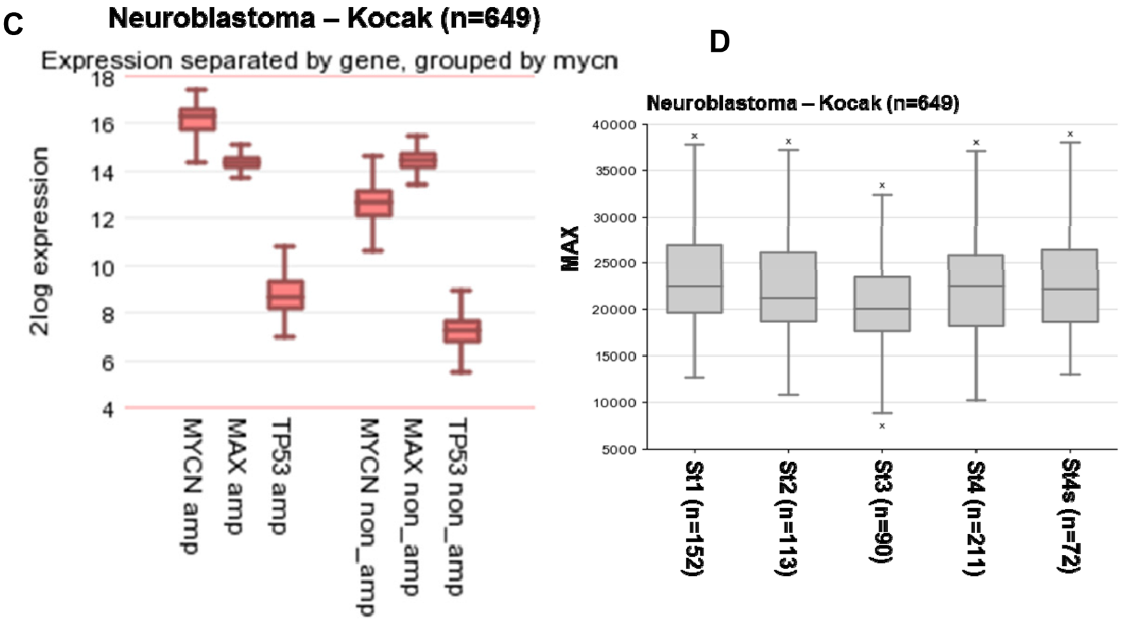

Supplementary Figure 8: Chromatin immunoprecipitation for MYC, MAX and p53 at the CDKN1A and CHEK1 loci using the IMR-32 MYCN amplified cell line. IMR-32 cells were not treated (control) or treated for 8h with 10 µM Nutlin-3a. ChIP was perfomed for MAX, MYCN, and p53. The fold enrichment was normalized using inputs and negative controls. **(A)** CDKN1A gene has both E-boxes and p53-Res. **(B)** CHEK1 does not have a canonical p53RE and q-PCR is for the MYCN binding E-box. All genes were analyzed using Chip-qPCR with appropriate primers listed in Supplementary Table 1. N3a indicates Nutlin-3a treatment. These data suggest that p53 activation does not lead to increased MAX binding at the loci associated with a marked increase in p53 chromatin binding. **(C)** Analysis of MYCN, MAX and p53 levels in MYCN amplified and non-amplified neuroblastoma in a large well annotated data set of patients by RNA-seq. MYCN levels are 4 log higher on average while MAX levels are basically unchanged between MYCN amplified and non-amplified cases. **(D)** MAX levels are relatively constant among all neuroblastoma stages (INSS staging). R2: Genomics Analysis and Visualization Platform (http://r2.amc.nl).

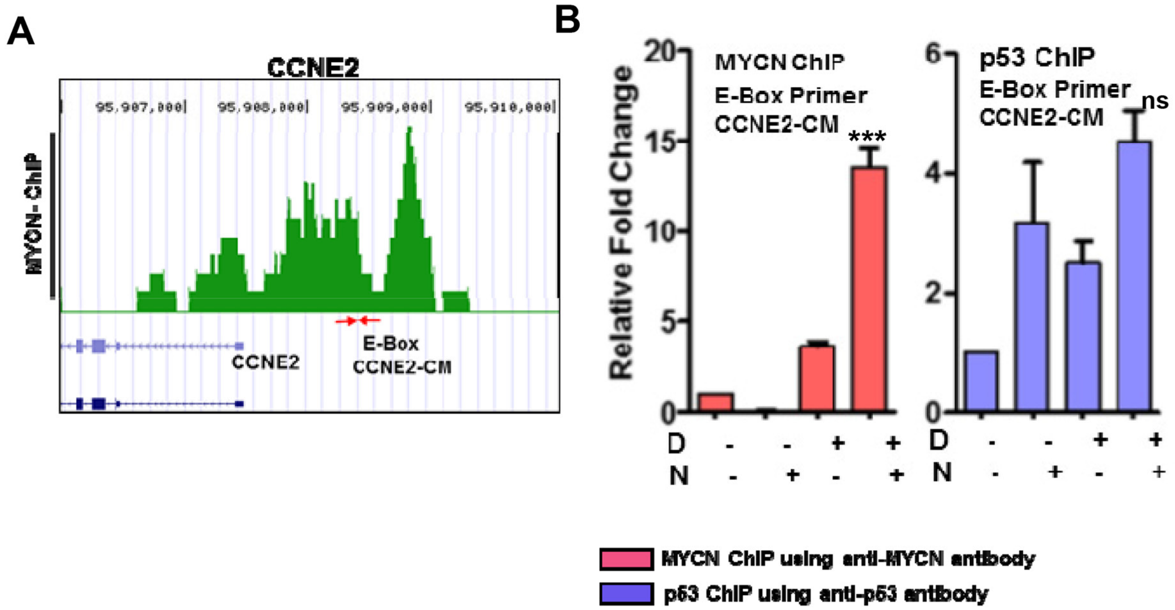

Supplementary Figure 9: Details of MYCN and p53 ChIP for the CCNE2 gene. **(A)** MYCN Chip-seq data across the 5' region upstream of the CCNE2 gene. Consensus E-box is high-lighted in red. **(B)** ChIP results for MYCN and for p53 using primers for the across the CCNE2 e-box site, demonstrating enrichment for both MYCN and p53 binding suggesting that p53 may form a complex with MYCN at this site. ChIP-qPCR primers design and ChIP-qPCR assays are as detailed in results section and in Figure 6. D = doxycycline for MYCN induction, N = nutlin treatment for p53 activation (see text).

Supplementary Table 1: Primers used in this study

Name[1]	Sequence (5'-3')
p21-CP-F	GATTGGCTTTCTGGCCGTCA
p21-CP-R	GGACAAAATAGCCACCAGCC
P21-CM-F	AGCTGGGTTAGGAACTTGGG
P21-CM-R	CGAAAACAAAACCGCTCGCA
SESN1-CP-F	TCGAGGGGGCATATCCACAG
SESN1-CP-R	GGCAGAAAATGCTAAGCTGGG
SESN1-CM-F	GCCCAGGTACCTCGTCCT
SESN1-CM-R	GTACACGGCCCCTTTGGC
CDC6-CM-F	CCCGCTTTACCCAGAGTCG
CDC6-CM-R	GAACGGGGGAGGGAATCTAC
CHEK1-CM-F	GGTCCGCCGTCCTTAAATCT
CHEK1-CM-R	TCCAAAAGGGTGACGTGGAG
CCNE2-CM-F	CCGGGATCCCAGAGGGTAA
CCNE2-CM-R	TGGTGGCGATCTTTCTTCCC
pGEX2T p53 1-93 F	AAGGATCCGAGGAGCCGCAGTCAGATCC
pGEX2T p53 1-93 R	TTGAATTCCAGGGGCCAGGAGGGGGCTGTGA
pGEX2T p53 full-length F	AAGGATCCGAGGAGCCGCAGTCAGATCC
pGEX2T p53 full-length R	TTGAATTCGTCTGAGTCAGGCCCTTCTGTCTGA
pGEX2T p53 1-363 F	TGACGA TCTGCCTCGCGCGTTTCG
pGEX2T p53 1-363 R	AGCCCTGCTCCCCCCTGGC
pGEX2T p53 FW 1-373 F	CTTTTTGGACTTCAGGTGGCTG
pGEX2T p53 FW 1-373 R	AGCCCTGCTCCCCCCTGGC
pGEX2T p53 1-383 F	GAGTTTTTTATGGCGGGAGGTA
pGEX2T p53 1-383 R	AGCCCTGCTCCCCCCTGGC
PET22B MYCN Full-length F	TTTCATATGCCGAGCTGCTCCACGTCC
PET22B MYCN Full-length R	TTTAAGCTTGCAAGTCCGAGCGTGTTC
pGEX2T MYCN 1-88-F	TTTGGATCCATGCCGAGCTGCTCCACGTCCACCAT
pGEX2T MYCN 1-88-R	TTTGAATTCGCTCGTTCTCAAGCAGCATCTCCGTG
pGEX2T MYCN 82-254-F	TTTGAATCCCGAGCTGGGTCACGGAGATGCTGCT
pGEX2T MYCN 82-254-R	TTTGAATTCGTACTGAGGGCCTTGTGGTCGCCGCC
pGEX2T MYCN 248-362-F	TTTGGATCCGACCACAAGGCCCTCAGTACCTCCG
pGEX2T MYCN 248-362-R	TTTGAATTCACACTCTTGAGCGGACGTGGGGACG CCTC
pGEX2T MYCN 336-400-F	TTTGGATCCTCTCCCTACGTGGAGAGTGAGGATGC
pGEX2T MYCN 336-400-R	TTTGAATTCGCTGGACCGAAGGTCGTTGCGGCGC
pGEX2T MYCN 400-464-F	TTTGGATCCAGCTTTCTCACGCTCAGGGACCACG TG
pGEX2T MYCN 400-464-R	TTTGAATTCCTAGCAAGTCCGAGCGTGTTCAATTTT CTTTA
pGEX2T c-MYC-F	TTTGGATCCGATTTTTTTCGGGTA
pGEX2T c-MYC-R	TTTGAATTCTTACGCACAAGAGTTCCG

[1]CP: ChIP p53; CM: ChIP MYCN.

Supplementary Table 2: Correlation of neuroblastoma patient outcome with modulated genes

See Supplementary File 1
