# Supplementary material for: MYCN acts as a direct co-regulator of p53 in MYCN amplified neuroblastoma
Source: Oncotarget. 2018 Apr 17;9(29):20323–38. doi: 10.18632/oncotarget.24859 (PMC5945521; doi:10.18632/oncotarget.24859)
Supplement: Supplementary file 2 [file oncotarget-09-20323-s002.docx]

**Supplementary Table 2:** Correlation of neuroblastoma patient outcome with modulated genes.

Correlation of neuroblastoma patient outcomes with genes modulated under MYCN high and p53 high conditions

| MYCN High and p53_High Downregulated genes | | | |
| --- | --- | --- | --- |
| Gene Name | p value | Expression | Outcome |
| FAM72B | 1.35E-38 | 5.06 | high |
| UNG | 3.97E-31 | 38.09 | high |
| SLBP | 5.88E-31 | 33.16 | high |
| DSCC1 | 6.33E-28 | 13.32 | high |
| SET | 1.16E-26 | 302.50 | high |
| SMARCA5 | 5.98E-26 | 118.46 | high |
| CDCA5 | 5.78E-25 | 51.53 | high |
| DTL | 1.06E-24 | 42.21 | high |
| HSPA14 | 2.95E-24 | 13.57 | high |
| WDR53 | 9.69E-24 | 6.30 | high |
| BUB1 | 1.65E-21 | 20.97 | high |
| ANP32E | 3.17E-21 | 52.38 | high |
| NUF2 | 1.26E-20 | 20.97 | high |
| CDCA7L | 9.17E-20 | 15.34 | high |
| DNA2 | 1.24E-19 | 18.34 | high |
| RFC1 | 7.52E-18 | 43.61 | high |
| KIAA1524 | 1.88E-17 | 17.94 | high |
| BRCA2 | 5.57E-16 | 12.00 | high |
| NUSAP1 | 3.82E-15 | 39.43 | high |
| ZWINT | 1.11E-14 | 36.65 | high |
| TRIM54 | 3.08E-14 | 4.26 | high |
| DCLRE1B | 6.60E-14 | 20.12 | high |
| C5ORF34 | 7.71E-14 | 6.68 | high |
| RRM2 | 8.95E-14 | 32.06 | high |
| XRCC3 | 1.42E-13 | 26.09 | high |
| FGD1 | 3.21E-13 | 45.23 | high |
| ZNF799 | 5.25E-13 | 5.98 | high |
| CDC20 | 2.25E-12 | 9.37 | high |
| CENPO | 3.18E-12 | 60.26 | high |
| ZNF670 | 6.65E-12 | 17.75 | high |
| DCLRE1A | 2.27E-10 | 25.59 | high |
| RFC5 | 1.74E-09 | 19.37 | high |
| CCDC77 | 3.66E-09 | 18.98 | high |
| SF3B2 | 6.77E-09 | 225.88 | high |
| ZNF738 | 2.02E-08 | 65.54 | high |
| FOXRED2 | 7.57E-07 | 51.81 | high |
| ARL13B | 9.03E-07 | 13.52 | high |
| IMPA2 | 9.41E-07 | 7.36 | high |
| ZNF700 | 2.64E-06 | 19.68 | high |
| PASK | 3.77E-06 | 35.13 | high |
| FKBP5 | 8.05E-06 | 75.91 | high |
| THAP7 | 3.31E-05 | 14.66 | high |
| ADORA1 | 6.86E-05 | 2.04 | high |
| ZNF703 | 1.03E-04 | 28.00 | high |
| WT1 | 6.36E-04 | 2.51 | high |
| CEP19 | 2.50E-03 | 10.10 | high |
| DBNDD2 | 3.67E-03 | 16.24 | high |
| RAD21 | 3.68E-03 | 172.37 | high |
| TTC7A | 2.61E-28 | 21.71 | low |
| KIAA1522 | 7.25E-25 | 22.45 | low |
| PQLC2 | 9.06E-22 | 6.78 | low |
| SPTBN1 | 6.95E-20 | 416.59 | low |
| DENND5B | 5.10E-15 | 49.81 | low |
| VCAM1 | 2.19E-14 | 6.63 | low |
| HEY1 | 5.93E-14 | 5.68 | low |
| HIP1 | 2.40E-11 | 60.55 | low |
| TFAP2A | 5.25E-10 | 1.50 | low |
| ID4 | 2.98E-08 | 3.99 | low |
| ZDHHC14 | 5.08E-08 | 9.94 | low |
| DYNLL2 | 7.68E-08 | 137.24 | low |
| EFNB2 | 2.33E-07 | 52.84 | low |
| SPIRE2 | 2.80E-07 | 31.11 | low |
| SIRPB1 | 4.03E-07 | 1.58 | low |
| ARID5A | 2.58E-06 | 6.73 | low |
| NR2F2 | 2.34E-05 | 19.14 | low |
| PIP4K2A | 3.21E-05 | 25.02 | low |
| PDE10A | 4.42E-05 | 36.56 | low |
| SALL1 | 2.18E-04 | 1.01 | low |
| MYH10 | 3.03E-04 | 91.45 | low |
| ADAP1 | 4.76E-03 | 2.79 | low |
| GNAZ | 5.94E-03 | 51.99 | low |
| DOPEY2 | 8.01E-03 | 32.74 | low |
| CDC42EP1 | 1.38E-02 | 11.41 | low |
| GLCE | 1.50E-02 | 13.06 | low |
| TCHP | 3.12E-02 | 20.84 | low |

| **MYCN High and p53_High Upregulated genes** | | | |
| --- | --- | --- | --- |
| **Gene Name** | **p value** | **Expression** | **Outcome** |
| TSKU | 1.17E-18 | 12.17 | high |
| ZNF561 | 1.41E-17 | 38.07 | high |
| RCL1 | 2.52E-15 | 14.46 | high |
| AMZ2 | 6.34E-12 | 50.39 | high |
| ARSG | 9.50E-11 | 3.42 | high |
| C12ORF66 | 6.40E-10 | 4.64 | high |
| PPM1D | 5.39E-09 | 54.00 | high |
| ZNF383 | 3.57E-07 | 6.66 | high |
| TSKU | 1.05E-05 | 24.44 | high |
| EPS8L2 | 2.43E-03 | 2.96 | high |
| STX12 | 8.20E-28 | 46.79 | low |
| MIB2 | 8.40E-23 | 32.98 | low |
| FCHO2 | 4.64E-17 | 22.86 | low |
| DGKA | 1.66E-15 | 5.59 | low |
| DNAJC16 | 2.72E-15 | 16.92 | low |
| EBI3 | 1.67E-14 | 1.73 | low |
| MAFK | 2.22E-13 | 8.27 | low |
| PTPN22 | 7.96E-13 | 1.53 | low |
| LSP1 | 1.15E-12 | 2.25 | low |
| CPE | 5.87E-12 | 117.49 | low |
| TSSK3 | 6.49E-12 | 3.68 | low |
| TMEM87B | 4.03E-11 | 14.97 | low |
| MED23 | 4.26E-11 | 32.82 | low |
| PGPEP1 | 9.57E-11 | 7.99 | low |
| ARHGEF3 | 1.36E-10 | 13.69 | low |
| STXBP3 | 5.94E-10 | 16.30 | low |
| ACER2 | 3.83E-09 | 3.29 | low |
| RETSAT | 5.95E-09 | 12.00 | low |
| YPEL5 | 4.66E-08 | 37.43 | low |
| SSPN | 8.83E-07 | 2.59 | low |
| XPR1 | 3.42E-06 | 43.67 | low |
| CSF1 | 4.16E-06 | 12.45 | low |
| REEP2 | 3.07E-05 | 92.14 | low |
| DRAM1 | 4.04E-05 | 4.24 | low |
| STX13 | 9.66E-06 | 24.29 | low |
| MIB3 | 1.01E-05 | 24.37 | low |
| RAB1A | 1.36E-03 | 64.03 | low |
| SYT1 | 4.68E-03 | 133.51 | low |
| PLCXD2 | 2.62E-02 | 2.28 | low |
